# The two phases of the Cambrian Explosion

**DOI:** 10.1038/s41598-018-34962-y

**Published:** 2018-11-09

**Authors:** Andrey Yu. Zhuravlev, Rachel A. Wood

**Affiliations:** 1 Department of Biological Evolution, Faculty of Biology, Moscow State University named after M.V. Lomonosov, Moscow GSP-1, 119991 Russia; 2 School of GeoSciences, University of Edinburgh, King’s Buildings, James Hutton Road, Edinburgh, EH9 3FE UK

## Abstract

The dynamics of how metazoan phyla appeared and evolved – known as the Cambrian Explosion – remains elusive. We present a quantitative analysis of the temporal distribution (based on occurrence data of fossil species sampled in each time interval) of lophotrochozoan skeletal species (n = 430) from the terminal Ediacaran to Cambrian Stage 5 (~545 – ~505 Million years ago (Ma)) of the Siberian Platform, Russia. We use morphological traits to distinguish between stem and crown groups. Possible skeletal stem group lophophorates, brachiopods, and molluscs (n = 354) appear in the terminal Ediacaran (~542 Ma) and diversify during the early Cambrian Terreneuvian and again in Stage 2, but were devastated during the early Cambrian Stage 4 Sinsk extinction event (~513 Ma) never to recover previous diversity. Inferred crown group brachiopod and mollusc species (n = 76) do not appear until the Fortunian, ~537 Ma, radiate in the early Cambrian Stage 3 (~522 Ma), and with minimal loss of diversity at the Sinsk Event, continued to diversify into the Ordovician. The Sinsk Event also removed other probable stem groups, such as archaeocyath sponges. Notably, this diversification starts before, and extends across the Ediacaran/Cambrian boundary and the Basal Cambrian Carbon Isotope Excursion (BACE) interval (~541 to ~540 Ma), ascribed to a possible global perturbation of the carbon cycle. We therefore propose two phases of the Cambrian Explosion separated by the Sinsk extinction event, the first dominated by stem groups of phyla from the late Ediacaran, ~542 Ma, to early Cambrian stage 4, ~513 Ma, and the second marked by radiating bilaterian crown group species of phyla from ~513 Ma and extending to the Ordovician Radiation.

## Introduction

The Cambrian Explosion is a phenomenon that encompasses the dramatic appearance of diverse metazoans with biomineralized skeletons, an increase in metazoan complexity and behaviour, a substrate revolution that re-organised the sedimentary record, and the development of biodiverse marine ecosystems with complex food webs^1–5^. The relative importance of external drivers, such as rise of oxygen or seawater chemistry changes^6–9^, biological drivers, such as the influence of metazoan irrigation^10^, and feedbacks between the two^11^, remains unclear. Likewise, the relationship between Ediacaran and Cambrian biotas remains unresolved, with some arguing that the Cambrian Explosion has a ‘deep root’ in the terminal Ediacaran^12^, or that the first phase of the ‘Cambrian Explosion’ was either the Nama assemblage (~550–541 Ma)^13^, or appeared even earlier at the Avalon-White Sea boundary at ~561 Ma^14^. In addition, while it has been conjectured that extinction or turnover events of metazoans occurred at ~551 Ma^13,15^ and at the Ediacaran/Cambrian boundary at ~541 - 540 Ma (e.g.^13,16^), there is no consensus as to the precise form either of these dynamics, or indeed their timing, or causes (compare^13,14,17^).

The combined body and trace fossil record suggests the Cambrian Radiation of bilaterians may have followed a progressive two-stage diversification: the terminal Ediacaran (~560 Ma) to early Cambrian Stage 2 to 3 (mid-Tommotian to Atdabanian) interval dominated by stem groups, and after Cambrian Stage 2 to 3 when definitive crown group representatives of phyla appeared^18^. Most phylum-level body-plan evolution seems to have taken place well after the Cambrian Explosion, throughout the Cambrian and beyond; stem lineages are considered to have largely disappeared by the Ordovician^18^.

Placing extinct fossil taxa in phylogenetic order through the application of stem- and crown group concepts allows the order of character acquisition to be considered in both time and environmental context^18^. Even when highly problematic, all extinct taxa must have stem- or crown group relationships to extant taxa. A *crown group* is a monophyletic group consisting of the last common ancestor of all living forms and all of its descendants. A *stem group* is a paraphyletic group that lacks the defining morphological characters of the crown group, where all members are extinct. This therefore consists of the primitive relatives of the crown group, along the phylogenetic line up to, but not including, the last common ancestor of the crown group and their nearest living relatives^19^.

The considerable number of characters that can define crown groups were often acquired incrementally over geological time^20^. Random, background extinctions will inevitably erode the base of a clade through time, whether or not basal members are particularly prone to extinction^19^. Hence, the older a fossil, the more likely it is to fall outside the phylum-level of classification. But mass extinctions may operate quite differently, as they can remove taxa selectively based on particular ecological or other traits^21^ and lead to long-lasting changes in taxonomic composition and ecosystem functioning^22^.

**Figure 1:**
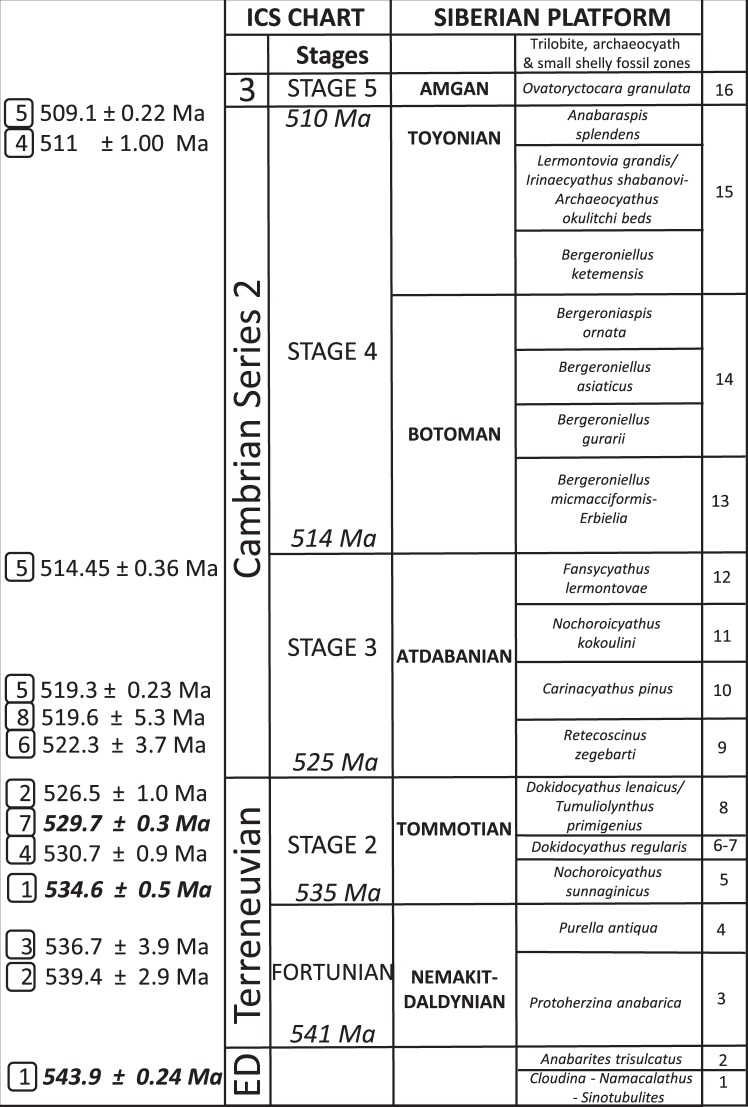
Early Cambrian time scale for the Siberian Platform, Russia, with key radiometric dates (numbered; Siberian radiometric dates are in bold), international chronostratigraphy (ICS), and stages and zones accepted for the Siberian Platform. Radiometric dates from 1^27,93^; 2^94^; 3^95^, 4^96^; 5^97^; 6^98–100^; 7^30^; 8^101^. Right column shows numbered temporal units, each c. 2.5 Myr in duration. ED = Ediacaran. 3 = Cambrian Series 3, pars. Modified from^5^.

Here we construct a high resolution temporal distribution of skeletal species (n = 1188) from the upper Ediacaran to the basal Cambrian Series 3 of the Siberian Platform in order to understand the evolutionary dynamics of the Cambrian Explosion (see Supplementary references). The Siberian Platform formed a separate province during the Ediacaran-Cambrian^23–26^, where the stratigraphy and age dating is relatively well known (Fig. 1) and the biota diverse. New coupled high-resolution δ^13^C and biostratigraphic data as well as improved U-Pb zircon dates suggests that terminal Ediacaran – early Cambrian sections on the northern and south-eastern Siberian Platform are more complete than previously thought, and also indicate that the Cambrian Explosion as shown by the record of skeletal biota may have been a more protracted event^12,27^. The first diverse skeletal assemblages of Cambrian type (including various halkieriids, chancelloriids, and hyoliths in addition to anabaritids and protoconodonts), occur between levels dated from 543.9 ± 0.24 to 529.7 ± 0.3 Ma which precede the strong basal Cambrian negative carbon isotope excursion (BACE), and in some areas even the basal Terreneuvian *Trichophycus pedum* ichnofossil assemblage^12,28–30^. Additionally, Ediacaran shelly taxa (cloudinids) co-occur with some of the earliest Cambrian shelly taxa (anabaritids) on the south-eastern Siberian Platform, indicating a continuity of the skeletal fossil record around the Precambrian-Cambrian boundary^12^. The additional presence of late Ediacaran soft-bodied rangeomorphs, including their biomineralized holdfasts, as well as chambered palaeopascichnids and *Nenoxites* (=*Shaanxilithes*) trace fossils found in immediately underlying strata of the same sections^12,27^ indicates that this record is, in turn, rooted in so-called “post-Kotlinian wormworld” (e.g.^13^).

In particular we consider the distribution of stem and crown group Lophotrochozoa, which is a monophyletic clade of protostome animals within the Spiralia, consisting of Mollusca, Lophophorata, Nemertea and Annelida^31–33^. The Lophotrochozoa constitutes a third of all modern marine animals^34^, and was chosen as it is species-rich and represented mostly by skeletal taxa in Ediacaran-Cambrian strata. Deuterostome and cnidarian fossils are too scarce for quantitative analysis, and putative poriferans do not allow detailed character subdivision, due to either an absence of diagnostic spicules (e.g. Archaeocyatha) or the frequently disarticulated preservation of spiculate classes. More importantly, neither the temporal fossil record nor comparative characters of the Lophotrochozoa are reliant upon exceptional preservation (Lagerstätten), as has been noted in other significant groups of the radiation such as euarthropods. This taphonomic bias is exemplified by the fact that crown group euarthropods appear before (521 Ma) stem lineage euarthropods (518 Ma), due in part to differential skeletonisation^35^. Our study thus enables an understanding of how important phyla including the Mollusca, Brachiopoda and Annelida, may have been assembled, in turn informing likely selective pressures and ecological consequences.

## Results

### Proposed stem-group Lophotrochozoa

The soft-bodied Ediacaran taxon *Kimberella* (~560 to ~550 Ma) has been proposed to represent a stem group mollusc^36–38^, although this placement remains problematic^17^. We exclude this from our analysis given this controversy and the lack of skeletonized hard parts.

We assign hyoliths (both hyolithimorphs and orthothecimorphs), tommotiids (including tannuolinids, *Sunnaginia*, and *Lapworthella*), and *Oymurania* to stem group lophophorates, and sachitids (including halkieriids and siphogonuchitids), wiwaxiids, and, probably, maikhanellids and helcionelloids to stem group molluscs following the phylogenetic and morphological inferences detailed below.

**Figure 2:**
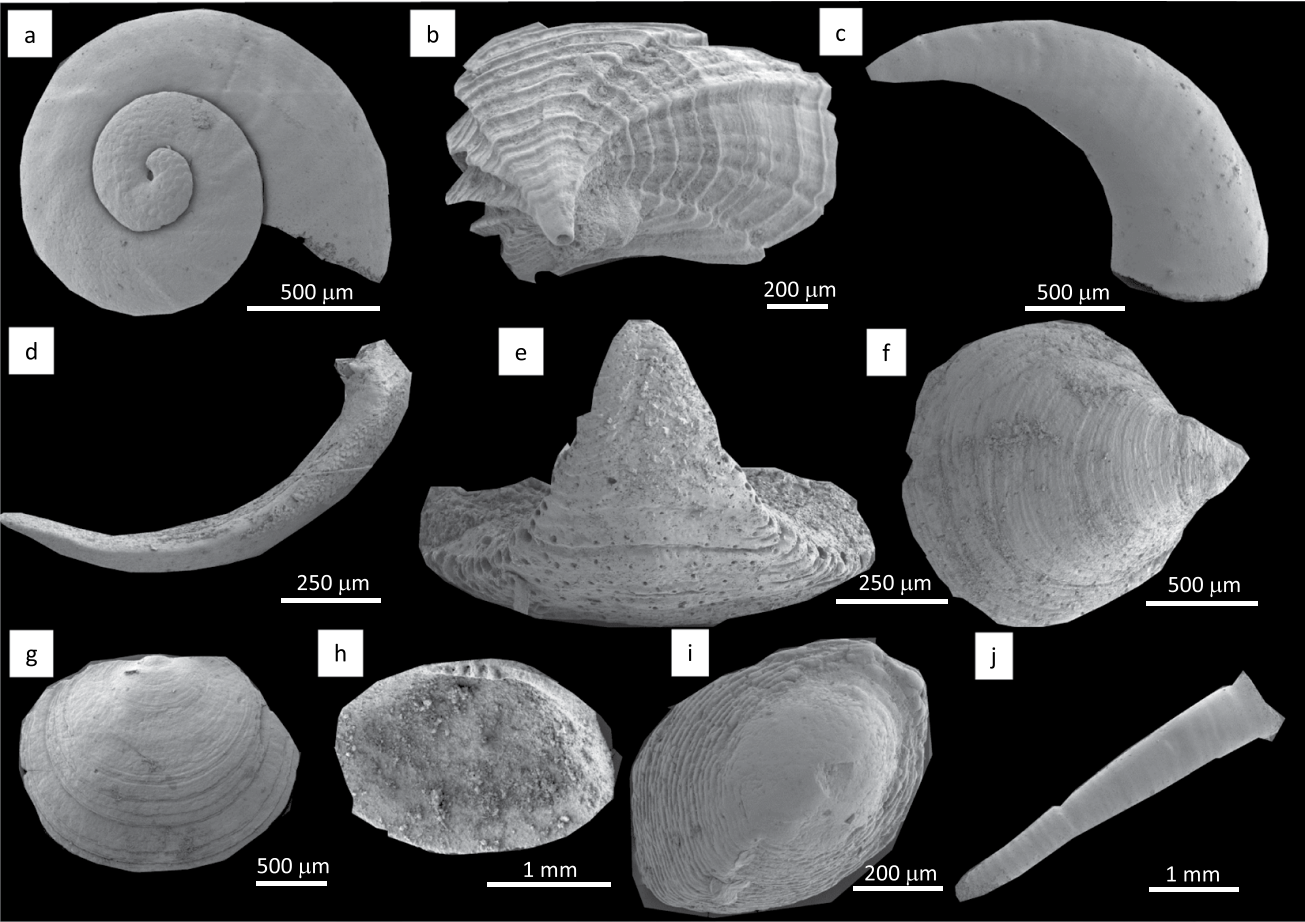
Early and early middle Cambrian skeletal stem- (**a**–**f**,**i**–**j**) and crown group (**g**,**h**) lophotrochozoans from the Siberian Platform. (**a**) *Aldanella attleborensis* (Shaler & Foerste), stem mollusc, helcionelloid; shell (^29^, Fig. 20A_1_); (**b**) *Camenella garbowskae* Missarzhevsky, stem lophophorate, tommotiid; sclerite (^102^, Fig. 37A); (**c**) *Ceratoconus striatus* Chen & Zhang, stem mollusc, helcionelloid; shell (^29^, Fig. 26A_1_); (**d**) *Halkieria* sp., stem lophotrochozoan; halkieriid; sclerite (^29^, Fig. 46C_2_); (**e**) *Tannuolina pavlovi* Kouchinsky *et al*., stem lophophorate, tommotiid; sclerite (^103^, Fig. 2A_2_); (**f**) *Oymurania gravestocki* Ushatinskaya, stem brachiopod; valve (^104^, Fig. 8A); (**g**) *Pelmanotreta neguertchenensis* (Pelman), crown brachiopod, paterinate; valve (^105^, Fig. 2i); (**h**) *Pojetaia dentifera* Kouchinsky *et al*., crown mollusc, bivalve; valve (^106^, Fig. 3A); (**i**) *Purella antiqua* (Abaimova), stem lophotrochozoan, maikhanellid; valve (^29^, Fig. 31B_2_); (**j**) *Khetatheca cotuiensis* (Sysoev), stem lophophorate, hyolith; valve (^29^, Fig. 50G). All photographs courtesy of Artem Kouchinsky.

Hyoliths (Fig. 2j), despite their unusual, large calcareous conical shells incorporating a U-shaped intestine and an extendable tentacle-bearing lophophore, have molluscan-type microstructures, a thick compound operculum and, sometimes in hyolithimorphs, a pair of additional curved rigid lateral bar-like supports^39,40^.

While both tommotiids (Fig. 2b,e) and halkieriids *s.l*. (Fig. 2d) possess multi-element shells (scleritomes), tommotiid sclerites form a narrow conical shell and penetrated by setal canals which can preserve phosphatized setae, and exhibit dense, and fine lamination. In some cases a bivalved larval protegulum with a colleplax-plate typical of the oldest linguliformean brachiopods is present^41–45^. *Oymurania* (Fig. 2f) has setigerous canals and two shell layers, one of which shows acrotretoid brachiopod columnar microstructure, and the other resembles the prismatic framework of paterinid brachiopods^46^. The former problematic fossil *Tumulduria* is now reinterpreted as a detached central portion of the ventral interarea of a paterinid brachiopod^47^.

Intact calcareous sachitid scleritomes are considered to belong to a bilateral motile organism that possessed a radula and sclerites with a branching, aesthete type of canal system found in some molluscs^48–50^. Complete sachitid scleritomes from the Early Ordovician are recognized as stem-group aculiferan molluscs^51^. Chancelloriid sclerites possess the same morphology and microstructure despite the presence of a markedly different scleritome of a sedentary radial-symmetrical animal^52–54^. Thus, a more basal position of sachitids among molluscs, or even lophotrochozoans, cannot be not excluded. Wiwaxiids, although being organic, show the same overall scleritome organization^55^.

The cup-shaped maikhanellids (Fig. 2i) consist of merged sclerites identical to co-occurring sachitids^56^. Their cross lamellar microstructure is similar to that of some gastropods and the cap-shaped protoconch is typical of monoplacophorans^57^. Bivalved calcareous stenothecoids with their paired, serially arranged muscle scars on the inner surfaces of both valves represent a further group of mollusc-like fossils but with a set of features uncommon in crown group molluscs^54^.

The majority of Cambrian mollusc-like shells with essentially molluscan microstructures, protoconchs, and some features of torsia, are assigned to either the class Helcionelloida^58^, or subclass Archaeobranchia^59^. These mainly cup-shaped and low spiral, endogastrically coiled fossils are considered to be extinct lineages of the phylum Mollusca^58,59^. However, a helcionelloid affinity suggests that their untorted anatomy is due to aperture posterior emarginations, and the presence of a snorkel in some forms also suggests that helcionelloids (Fig. 2a,c) occupy a basal position within the phylum. The archaeobranchian hypothesis also emphasises a torted basic plan and ancestral gastropod affinities^59^. A stem group rather than crown group position for helcionelloids is further supported by the presence of paired bristle-like clusters extending from the aperture of the *Pelagiella* shell which have a striking resemblance to the parapodial chaetae of some polychaetes^60^. Additionally, helcionelloids are characterized by a different muscle system, densely porous shells that are more common in brachiopods than molluscs, and calcitic semi-nacre microstructures which are more typical of lophophorates^61–63^. These observations suggest that the Helcionelloida were stem-group molluscs that retained a number of shared basal features with lophotrochozoan ancestors. *Pelagiella* represents the most advanced branch of helcionelloids possessing a spirally coiled shell and asymmetric muscle scars suggesting at least partial torsion^59^.

### Proposed crown-group Lophotrochozoa

Early Cambrian crown-group molluscs (Fig. 2h), are recognized among bivalves as well as gastropods of the Khairkhaniidae and Onychochilidae families, belonging to the Divasibranchia and Dextrobranchia orders, respectively^59,64^. Crown group Lophophorata are represented in the Cambrian by 13 orders of brachiopods (Fig. 2g), only one of which (Lingulida) survived beyond the Palaeozoic^45,65,66^.

### Quantitative temporal distribution

**Figure 3:**
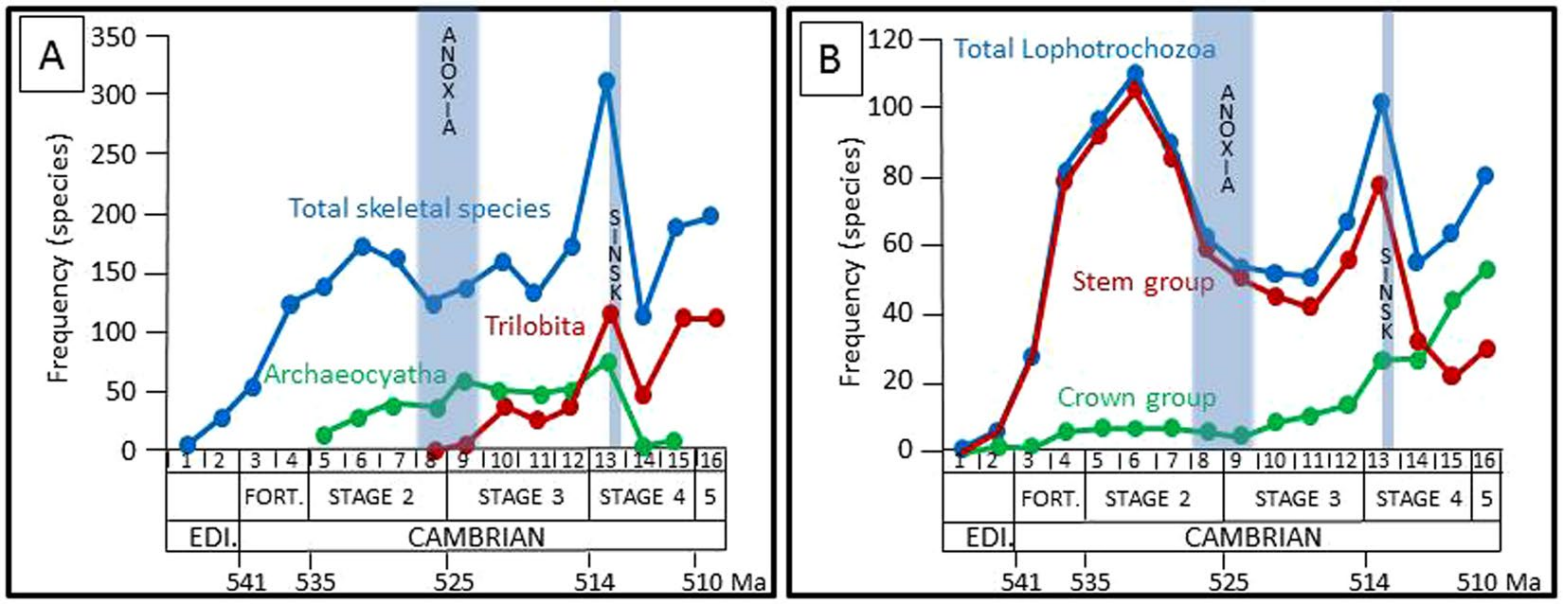
Diversity of skeletal species through the Ediacaran – early Cambrian of the Siberian Platform. (**A**) Total diversity of all skeletal species, Trilobita, and Archaeocyatha. (**B**) Total diversity of skeletal lophotrochozoan species, and stem group and crown group representatives. Ediacaran and Cambrian chronostratigraphic subdivisions are scaled according to Fig. 1.

Total skeletal species diversity on the Siberian Platform increases from the terminal Ediacaran to the middle of Cambrian Stage 2, then declines and rises again to reach a second peak at the beginning of Stage 4, followed by an abrupt and rapid decline at the end of Stage 4, followed by recovery around the Series 2/3 boundary (Fig. 3A). The Trilobita appears in Stage 3 and, as the most speciose group, mirrors this general trend. This is in contrast to the second most speciose group, the Archaeocyatha, which first appears in Stage 2 after which there is increase in diversity until the base of Stage 4 but then the group goes abruptly extinct shortly thereafter (Fig. 3A).

Total skeletal lophotrochozoan species diversity likewise increases from the terminal Ediacaran to the middle of Cambrian Stage 2, but then declines until the middle of Stage 3, rises again to reach a second peak at beginning of Stage 4, followed by an abrupt and rapid decline until the middle of Stage 4, then followed by a further rise (Fig. 3B).

Of these, stem group lophophorates, brachiopods, and molluscs comprise a total of 354 species, and crown-groups a total of 76 species through the Ediacaran to Cambrian Stage 5 interval. Stem lophoporates, brachiopods and molluscs (halkieriids, chancelloriids and orthothecimorph hyoliths) appeared in the terminal Ediacaran (~542.5 Ma) and show two phases of diversification: the first through the Terreneuvian, and the second during the end of Stage 3 to beginning of Stage 4 (Fig. 3B). The first crown species are known from the late Fortunian (~537 Ma – gastropods; ~535 Ma – brachiopods and bivalves), and started to radiate later during the early Cambrian Epoch 2 (~522.5 Ma). Stem group species were devastated during the early Cambrian Stage 4 at ~513 Ma but crown group mollusc and brachiopod species, despite some changes in species composition, show no marked loss of diversity, and continued to diversify at a similar apparent rate (Fig. 3B).

## Discussion

### Possible taphonomic and sampling biases

Taphonomic studies have shown that the fossil record can test the proposition that marine community structure has changed over time^67,68^. Ediacaran to Cambrian skeletal lophotrochozoans are represented by taxa of comparable millimetric sizes, forming part of the small shelly fauna as shells and disarticulated sclerites. These fossils are generally either replaced by phosphate or present in the form of inner and outer moulds. Only lingulate brachiopods and tommotiids are preserved as original shells, and only rhynchonelliform brachiopods retain their original low-Mg calcite mineralogy. In the lower Cambrian of the Siberian Platform, such fossils are restricted to argillaceous limestones (mostly wackestones and packstones), and some grainstones, all of which accumulated onshore above either normal wave or storm wave base^69^. All fossils are extracted by the same method of dissolution in buffered acetic acid to isolate phosphatic and phosphatized shells, or moulds and steinkerns (e.g.^29^). Worker bias is unlikely given that the assemblages reflect multiple different studies and no single worker or study dominates. We infer that taphonomic biases are minimized, and sampling biases present are shared by all small skeletal fossils.

### Trends through time

Total lophotrochozoan biodiversity increases until the middle of Stage 2, but then there is a notable decline that extends to approximately the middle of Stage 3 (Fig. 3B). This interval coincides in part with an expansion of anoxic sea floor around ~525 Ma inferred from U isotopes^70^. Stem- and crown group lophotrochozoan species show distinctly different temporal distributions, with stem group lophophorate, brachiopod and mollusc taxa originating and radiating first. The preferential extinction of stem group species in early Cambrian Stage 4, at ~513 Ma coincides with the well-known Sinsk Event, an episode of widespread shallow marine anoxia on the Siberian Platform and other locations globally, which also coincides with the major extinction of the Archaeocyatha^71^. It is probable that Archaeocyatha represent a poriferan stem group, and indeed a similar temporal separation of stem and crown group diversification is observed among other metazoans at phyla level, including the Porifera (where crown group demosponges are known by Cambrian Stage 3), Cnidaria and Echinodermata^54,72–74^.

The first probable metazoan body fossils (rangeomorphs) appeared at ~570 Ma^75^. Rangeomorphs are complex, macroscopic eukaryotes, probably stem group metazoan taxa, although an affinity higher than Porifera has been proposed^76^. Rangeomorph-dominated assemblages were devastated by the Kotlin Crisis, which marks a turnover event^15^. After this we propose two phases of the Cambrian Explosion separated by the Sinsk Event extinction. The first was dominated by non-bilaterians (Porifera, Cnidaria and Ctenophora) joined by indeterminate bilaterian stem groups at ~ 560 Ma^18^ and lasted until ~513 Ma. The general increase in diversity may have been interrupted by the global expansion of anoxic sea floor around ~525 Ma. Notably, this diversification started before, and continues across, the Ediacaran/Cambrian boundary and the Basal Cambrian Carbon Isotope Excursion (BACE) interval (~541 to ~540 Ma). The BACE has been ascribed to a possible global perturbation of the carbon cycle^12^.

**Figure 4:**
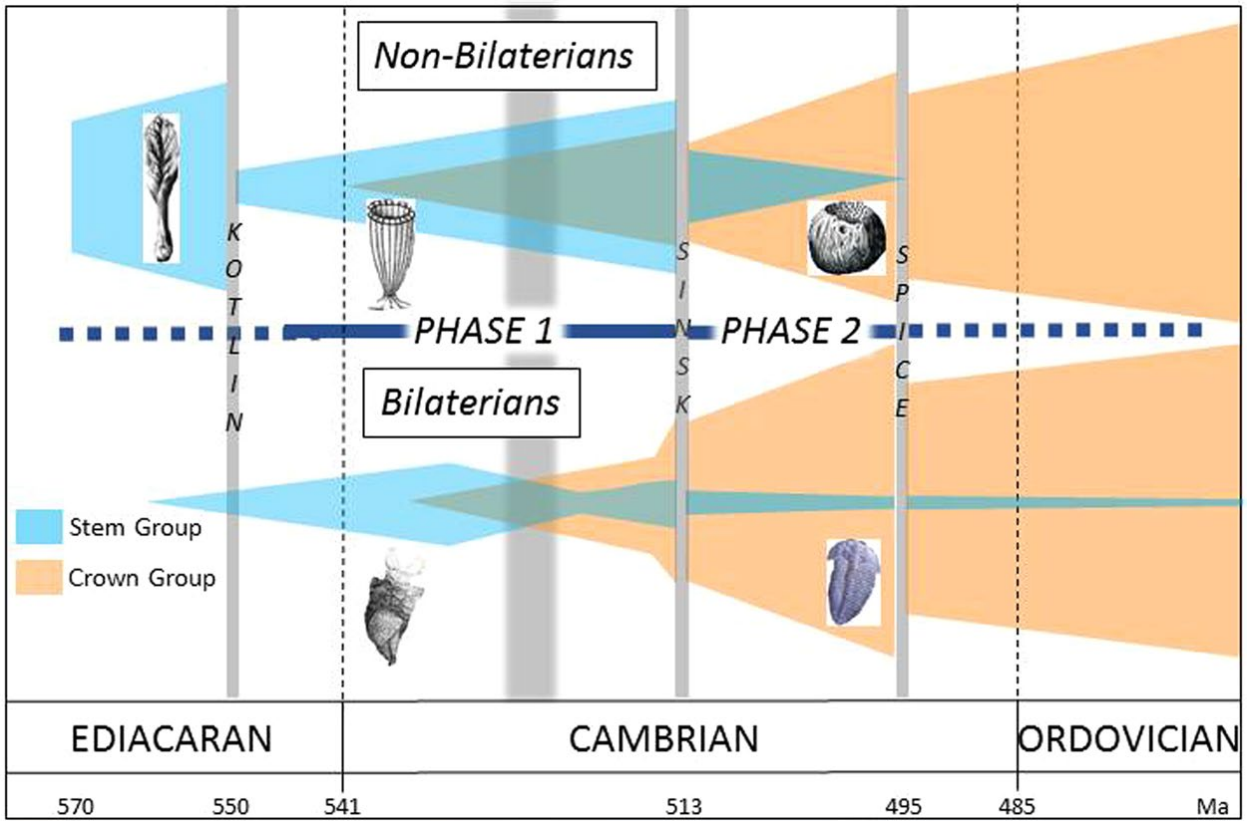
Schematic of hypothesised non-Bilaterian (total group Porifera, Cnidaria and Ctenophora) and Bilaterian diversification during the Ediacaran-Cambrian metazoan radaition, showing the fossil record of probable earliest metazoans (shown by a rangeomorph reconstruction), the Kotlin crisis, followed by two phases of Cambrian Explosion, separated by the Sinsk Event extinction (with a possible expanded interval of anoxia during Phase 1) and extending to the Ordovician Radiation through the SPICE extinction. Non-bilaterian stem group example is a stem group archaeocyath sponge; crown group is a crown group demosponge. Bilaterian stem group is shown by a tommotiid; crown group by a trilobite.

The second phase was marked by radiating non-bilaterian and bilaterian (here determined as brachiopod and mollusc) crown group species, and started from ~513 Ma. This second radiation phase may have been interrupted or even terminated by the late Cambrian SPICE event, which marked a further minor extinction (Fig. 4). Crown groups brachiopod species continued to diversify during the remainder of the Cambrian and into the Ordovician. Bivalves and gastropods also formed a significant part of total global lophotrochozoan diversity and were joined by the appearance of bryozoans and cephalopods around the Cambrian/Ordovician boundary^77,78^. From that time onwards their diversity remained higher than stem group lophotrochozoans, which continued to decline dramatically during the Cambrian^51,59,71,79,80^. The last stem group taxa (a few hyolith genera) went extinct in the Permian^81^.

The Sinsk Event might therefore be considered a mass extinction, which appears to have preferentially removed skeletal stem group lophotrochozoans at a point when diversity was high. This rapid removal is in contrast to background extinctions that are expected to erode the base of a clade gradually through time. We note that crown group brachiopod and mollusc do not show a marked increase in diversity after the removal of stem group lophophorate, brachiopod and mollusc taxa, but continue their former diversity trajectory. This suggests that their radiation was not dependent upon the removal of incumbent stem group taxa, but rather that crown group taxa were in some way more resilient to shallow marine anoxia or other coeval environmental perturbations. Like other mass extinctions, the Sinsk Event led to significant and long-lasting changes in taxonomic composition and ecosystems^22,79,82^.

A similar sequential faunal replacement pattern of Phanerozoic metazoans has been established in the form of evolutionary marine faunas^83^, which are in part bounded by mass extinctions. During the Ediacaran to Cambrian interval, further distinguished were the Tommotian, Cambrian *s.s*. and Palaeozoic faunas^84^. All these faunas were discriminated by empirical and statistical analysis of family diversity patterns only without reference to phylogenetic relationships. Their existence was, however, challenged^22^ because their speciation/extinction trends could merely reflect replacement between major taxonomic groups that had coupled dynamics. But our analysis shows that evolutionary faunas may in fact be a manifestation of their composition, with the ‘Tommotian’ fauna being composed of mostly stem group lophotrophorates, molluscs and brachiopods, while the Cambrian *s.s*. and Palaeozoic faunas are dominated by crown group representatives of molluscs, brachiopods and many other phyla.

This pattern resembles the extinction of taxa at the Permo-Triassic boundary, when groups that originated in the early Palaeozoic either went extinct (tabulate and rugose corals, trilobites, cystoporates) or significantly declined (brachiopods, trepostomates, cryptostomates, conodonts) never to recover previous levels of diversity^85^. This is in contrast to the pattern shown by groups which appeared and diversified in the late Palaeozoic, such as gymnolaemates and new bivalve, gastropod and ammonoid orders^85–87^.

If ecological niches are relevant, the difference in maintaining the two phases of the Cambrian Explosion might be related to differences in ecospace that was actually “empty” for skeletal animals. During the earlier phase of stem taxa radiation (~543–513 Ma), speciation was most likely promoted by the lack of competition for existing niches. This is similar to the high rates of sympatric speciation, such as noted among modern benthic caenogastropods in lakes, where high phenotypic plasticity enables evolving ecophenotypes to diversify into different substrates (e.g.^88^). A similar pattern of early diversification as a result of adaptations to different substrates is shown by both helcionelloid mollusc and archaeocyath sponge species during the first phase of the Cambrian Explosion inferred here. The helcionelloids underwent rapid morphogenesis^89^, and archaeocyaths display extremely high inter-habitat diversity (that is, beta-diversity) in reef communities on the Siberian Platform^90^, which may also reflect high speciation rates. Niche partitioning is not inferred, as the alpha-diversity (species number per community) remains consistently low^91^. A similar effect as a result of low competition, and also correlated with a rise in beta-diversity, has been observed to be the main driver of general diversity increase in the early Cambrian^92^. This dynamic creates the unusual situation when the boundaries of even major lower Cambrian subdivisions have not been established due to an absence of cosmopolitan species. By contrast, even though stem group diversity was significantly reduced during the later crown group brachiopod and mollusc diversification (~513–508 Ma), older niches were not completely eliminated. Thus, in the aftermath of the Sinsk extinction, crown groups were able to diversify via competition for existing niches in order to incorporate into existing communities.

## Conclusions

This quantitative analysis of lophotrochozoan skeletal stem- and crown group temporal distribution suggests that the Cambrian Explosion sensu lato may be redrawn as two successive phases of morphological and functional innovation that started in the terminal Ediacaran and were separated by an extinction event. This in turn allows exploration of this phenomenon as an expansion of ecological repertoires that are tractable from the fossil record.

## Methods and Data

We divide the terminal Ediacaran to Cambrian Series 2 Siberian record from ~545 to ~505 Ma based on radiometric dates into 16 temporal units based on either sub-division, or combination of one to three Siberian biostratigraphic zones to create broadly equivalent units of ~2.5 Myr each. Units start at the Ediacaran *Cloudina-Namacalathus-Sinotubulites* assemblage zone through transitional Ediacaran-lowermost Cambrian zones (informally named in ascending order *Anabarites trisulcatus, Protohertzina anabarica*, and *Purella antiqua* zones) through Terreneuvian and Cambrian Series 2 zones up to the basal *Ovatoryctocara granulata* Zone of the Cambrian Stage 5 (Series 3) (Fig. 1). We use the timescale for this interval from available radiometric dates from fossiliferous strata of Siberia, South China, and Avalonia^93–101^ (see Supplementary data).

We quantify the distribution of described skeletal species (n = 1188) from the upper Ediacaran to the basal Cambrian Series 3 on the Siberian Platform (see Supplementary references). This is derived from occurrence data of fossil taxa sampled in each time interval. In particular we quantify the temporal distribution of lophotrochozoan skeletal species (n = 430) (see Supplementary data). Chancelloriids, although they may belong to stem lophotrochozoans, are excluded from analysis due to their frequently disarticulated nature.

## Electronic supplementary material

Supplementary Information

## Data Availability

The authors declare that the data supporting the findings of this study are available within the paper and its supplementary information files.
