# Supplementary material for: The two phases of the Cambrian Explosion
Source: Sci Rep. 2018 Nov 9;8:16656. doi: 10.1038/s41598-018-34962-y (PMC6226464; doi:10.1038/s41598-018-34962-y)
Supplement: Supplementary file 1 — Supplementary Information [file 41598_2018_34962_MOESM1_ESM.doc]

**The two phases of the Cambrian Explosion**

**Andrey Yu. Zhuravlev^1^, Rachel A. Wood^2*^**

**^1^**Department of Biological Evolution, Faculty of Biology, Moscow State University named

after M.V. Lomonosov, Moscow GSP-1, 119991, Russia

**^2^**School of GeoSciences, University of Edinburgh, King's Buildings, James Hutton Road,

Edinburgh EH9 3FE, UK. [*Rachel.Wood@ed.ac.uk](mailto:*Rachel.Wood@ed.ac.uk)

****Supplementary Information and Data****

**Supplementary Information and Data**

1. **References for quantitative temporal distribution of skeletal taxa on the Siberian platform**

Aksarina, N.A. & Pel’man, Yu.L. 1978. (Cambrian Brachiopods and Bivalved Molluscs of Siberia). Institut Geologii i Geofiziki Sibirskogo Otdeleniya Akademii Nauk SSSR, Trudy 316, 1–178 (in Russian).

Andreeva, O.N. 1987. (Cambrian artriculate brachiopods). Paleontologicheskiy zhurnal 1987 (4): 31–40 (in Russian).

Andreeva, O.N. 1989. (On some Cambrian-Ordovician brachiopods of the Siberian Platform). Ezhegodnik Vsesoyuznogo Paleontologicheskogo Obshchestva 32: 4–76 (in Russian).

Astashkin, V.A., Pegel’, T.V., Repina, L.N., Rozanov, A.Yu., Shabanov, Yu.Ya., Zhuravlev, A.Yu., Sukhov, S.S. & Sundukov, V.M. 1991. The Cambrian System on the Siberian Platform. Correlation chart and explanatory notes. International Union of Geological Sciences, Publication 27, 1–133.

Astashkin, V.A., Varlamov, A.I., Egorova, L.I. & Shabanov, Yu.Ya. 1981. (Stratigraphic position of trilobites of the “Sanashtykgol” assemblage in the stratotype section of the Lower Cambrian of the Lena River). Sibirskiy Nauchno-Issledovatel’skiy Institut Geologii, Geofiziki i Mineral’nogo Syr’ya, Trudy 287, 5–15 (in Russian).

Barskova, M.I. 1987. (New species of Lower Cambrian gastropods from the Uchur-Maya region). Paleontological Zhurnal 1987 (2), 124–127 (in Russian).

Barskova, M.I. 1988. (New molluscs from the Lower Cambrian strata of the Kolyma Uplift). Paleontological Zhurnal 1988 (1), 101–105 (in Russian).

Bengtson, S. 1970. The Lower Cambrian fossil *Tommotia*. *Lethaia* 3, 363–392.

Bengtson, S. 1977. Early Cambrian button-shaped phosphatic microfossils from the Siberian Platform. Palaeontology 20, 751–762.

Bengtson, S., Matthews, S.C. & Missarzhevsky, V.V. 1986. The Cambrian netlike fossil *Microdictyon*. In A. Hoffman & M.H. Nitecki, eds. Problematic Fossil Taxa, pp. 97–115. New York, Oxford University Press; Oxford, Clarendon Press.

Bengtson, S., Fedorov, A.B., Missarzhevsky, V.V., Rozanov, A.Yu. & Zhuravlev, A.Yu. 1987. *Tumulduria incomperta* and the case for Tommotian trilobites. Lethaia 20, 361–370.

Bokova, A.R. 1985. (The oldest assemblage of Cambrian organisms of western Prianabar’e). In V.V. Khomentovsky, ed. (Stratigraphy of the Late Precambrian and Early Palaeozoic of Siberia. Vendian and Riphean), pp. 13-28. Novosibirsk, Institut Geologii i Geofiziki Sibirskogo Otdeleniya Akademii Nauk SSSR (in Russian).

Bokova, A.R. 1990. (New Lower Cambrian gastropods of the Siberian Platform). Paleontological Zhurnal 1990 (2), 123–126 (in Russian).

Bushuev, E., Goryaeva, I. & Pereladov, V. 2014. New discoveries of the oldest trilobites *Profallotaspis* and *Nevadella* in the northeastern Siberian Platform, Russia. Bulletin of Geosciences 89, 347–364.

Chernysheva, N.E. 1950. [Cambrian Strata of the Upper Priangar’e, Their Fauna and Position in the Generalised Section of the Cambrian of the Central Parts of the Siberian Platform (Yakutia)]. Irkutsk, Vostsibneftegeologiya. 44 p. (in Russian).

Chernysheva, N.E. 1961. (Stratigraphy of the Cambrian of the Aldan Anteclise and the palaeontological grounds for distinguishing the Amgan Stage). Vsesoyuzniy Nauchno-Issledovatel’skiy Geologicheskiy Institut, Trudy (Novaya Seriya) 49, 1–347 (in Russian).

Chernysheva, N.E. 1962. (Cambrian trilobites of the family Oryctocephalidae). Nauchno-Issledovatel'skiy Institut Geologii Arktiki, Trudy 127, pp. 3–52 (in Russian).

Datsenko, V.A., Zhuravleva, I.T., Lazarenko, N.P., Popov, Yu.N. & Chernysheva, N.E. 1968. (Biostratigraphy and fauna of the Cambrian deposits of the northwestern Siberian Platform). Nauchno-Issledovatel’skiy Institut Geologii Arktiki, Trudy 155, 1–213 (in Russian).

Debrenne, F., Lafuste, J. & Zhuravlev, A. 1990. Coralomorphes et spongiomorphes a l'aube du Cambrien. Bulletin du Muséum national d‘Histoire naturelle, Paris, 4e série 12, 17–39.

Debrenne, F., Rozanov, A. & Zhuravlev, A. 1990. Regular Archaeocyaths. Éditions du Centre National de la Recherche Scientifique, Cahiers de Paléontologie. Paris. 218 p.

Debrenne, F, & Zhuravlev, A. 1992. Irregular Archaeocyaths. Éditions du Centre National de la Recherche Scientifique, Cahiers de Paléontologie. Paris. 212 p.

Debrenne, F., Zhuravlev, A.Yu., & Rozanov, A.Yu. 1988. (New genera of regular tabular and single-chambered archaeocyaths from the Lower Cambrian of Siberia). Paleontologicheskiy Zhurnal 1988 (4), 97–99 (in Russian).

Demidenko, Yu.E. 2006. New Cambrian lobopods and chaetognaths of the Siberian Platform. Paleontological Journal 40, 234–243.

Demidenko, Yu.E. 2016. Morphology, taxonomic position, and stratigraphic distribution of the Early Cambrian skeletal problematic *Mobergella radiolata* Bengtson, 1968. Paleontological Journal 50, 435–449.

Demokidov, K.K. & Lazarenko, N.P. 1959. (New data on the stratigraphy of Cambrian strata of the western slope of the northern Kharaulakh), In N.A. Shvedov, ed. (Collection of Papers on Palaeontology and Stratigraphy, Issue 16), pp. 11–22. Leningrad, NIIGA (in Russian).

Demokidov, K.K. & Lazarenko, N.P. 1964. (Upper Precambrian and Cambrian stratigraphy and the Lower Cambrian trilobites of the northern part of Middle Siberia and the Soviet Arctic islands), Nauchno-Issledovatel'skiy Institut Geologii Arktiki, Trudy 137, 1–288 (in Russian).

Dzik, J. 1994. Evolution of ‘small shelly fossils’ assemblages. Acta Palaeontologica Polonica 39, 247–313.

Egorova, L.I. 1967. (Some Lower and Middle Cambrian trilobites of the Siberian Platform). Paleontologicheskiy Zhurnal 1967 (1), 68–78 (in Russian).

Egorova, L.I. 1970a. (New Middle Cambrian trilobites of the north of the Siberian Platform). Paleontologicheskiy Zhurnal 1970 (4), 72–76 (in Russian).

Egorova, L.I. 1970b. (Cambrian trilobites of the east of the Siberian Platform and their facies restriction). Sibirskiy Nauchno-Issledovatel’skiy Institut Geologii, Geofiziki i Mineral’nogo Syr’ya, Seriya Regional’naya Geologiya, Trudy 110, 39–46 (in Russian).

Egorova, L.I. 1983. (New Lower Cambrian trilobites of the southeastern Siberian Platform). Paleontologicheskiy Zhurnal 1983 (3), 59–64 (in Russian).

Egorova, L.I. & Korobeynikova, T.V. 1974. (Trilobite assemblages of the type sections of the Obruchev and Elanka horizons from the Lower Cambrian of Siberia). Sibirskiy Nauchno-Issledovatel’skiy Institut Geologii, Geofiziki i Mineral’nogo Syr’ya, Trudy 192, 35–45 (in Russian).

Egorova, L.I. & Savitskiy, V.E. 1969. (Cambrian stratigraphy and biofacies of the Siberian Platform. Western Anabar area). Sibirskiy Nauchno-Issledovatel’skiy Institut Geologii, Geofiziki i Mineral’nogo Syr’ya, Trudy 43, 1–408 (in Russian).

Egorova, L.I. & Shabanov, Yu.Ya. 1987. (Cambrian trilobites on the north of the Anabar-Sinsk facies region of the Siberian Platform). In S.P. Bulynnikova & I.G. Klimova, eds. (New Species of Ancient Plants and Invertebrates from the Phanerozoic of Siberia), pp. 69–79. Novosibirsk, SNIIGGiMS (in Russian).

Egorova, L.I., Shabanov, Yu.Ya. & Evtushenko, V.M. 1969. (On the stratigraphy of the Perekhod and Sinsk formations in the Lenan Stage stratotype of the Lower Cambrian). Sibirskiy Nauchno-Issledovatel’skiy Institut Geologii, Geofiziki i Mineral’nogo Syr’ya, Trudy, Seriya Regional’naya Geologiya 84, 11–20 (in Russian).

Egorova, L.I., Shabanov, Yu.Ya., Rozanov, A.Yu., Savitskiy, V.E., Chenysheva, N.E. & Shishkin, B.B. 1976. (Elanka and Kuonamka facies stratotypes of the lower boundary of the Middle Cambrian in Siberia). Sibirskiy Nauchno-Issledovatel’skiy Institut Geologii, Geofiziki i Mineral’nogo Syr’ya, Trudy 211, 1–167 (in Russian).

Fedorov, A.B. 1982a. (Biostratigraphy and facies of the oldest Lower Cambrian horizon in the middle reaches of the Aldan River). In: V.A. Astashkiv, ed. (Stratigraphy and Facies of Sedimentary Basins of Siberia), pp. 18–25. Novosibirsk, SNIIGGiMS (in Russian).

Fedorov, A.B. 1982b. [Precambrian and Cambrian boundary strata in the south of the Siberian Platform (analysis of facies conditions)]. In: V.I. Krasnov, ed. (Boundaries of Principal Phanerozoic Subdivision in Siberia), pp. 29–39. Novosibirsk, SNIIGGiMS (in Russian).

Fedorov, A.B. 1984. [New representatives of skeletal organics in the Precambrian-Cambrian stratotype sections of the Siberian Platform (Aldan, Kotuy rivers)]. In S.P. Bulynnikova & I.G. Klimova, eds. (New Species of Ancient Invertebrates and Plants from Oil-Gas-Bearing Provinces of Siberia), pp. 5–9, 89–90. Novosibirsk, SNIIGGiMS (in Russian).

Fedorov, A.B. 1986. (New tubicolous problematics from the Tommotian Stage stratotype). Paleontologicheskiy Zhurnal 1986 (2), 110–111 (in Russian).

Fedorov, A.B. & Shishkin, B.B. 1984. (Lower boundary of the Cambrian in the north of the Siberian Platform). In: V.I. Krasnov, ed. (Problems of the Stage Subdivision of the Phanerozoic Systems in Siberia), pp. 5–14. Novosibirsk, SNIIGGiMS (in Russian).

Golubev, S.N. 1976. (Ontogenic changes and evolutionary trends in the Early Cambrian spiral gastropods Pelagiellacea). Paleontologicheskiy Zhurnal 1976 (2), 34–40 (in Russian).

Goryaeva, I.E. 2010. (Trilobites from the Keteme Formation of the Toyonian Stage, Lower Cambrian of the Siberian Platform]. In I.V. Budnikov & B.G. Kraevsky, eds. (Regional Geology, Stratigraphy and Palaeontology of the Precambrian and Lower Palaeozoic of Siberia], pp. 53–74. Novosibirsk, SNIIGGiMS (in Russian).

Goryanskiy, V.Yu. 1977. (New Early Cambrian obollelids of eastern Siberia). In G.A. Stukalina, ed (New Species of Ancient Plants and Invertebrates of the USSR, Issue 4), pp. 99–102. Moscow, Nauka (in Russian).

Goryanskiy, V.Yu., Egorova, L.I. & Savitskiy, V.E. 1964. (On the Lower Cambrian fauna of the northern slope of the Anabar Shield). In N.A. Shvedov, ed. (Scientific Reports. Series Palaeontology and Biostratigraphy, Issue 4), pp. 5–32. Leningrad, NIIGA (in Russian).

Gubanov, A.P., Kouchinsky, A.V., Peel, J.S. & Bengtson, S. 2004. Middle Cambrian molluscs of ‘Australian type’ from northern Siberia. Alcheringa 28, 1–20.

Gubanov, A.P. & Peel, J.S. 1999. *Oelandiella*, the earliest Cambrian helcionellod mollusc from Siberia. Palaeontology 42, 211–222.

Gubanov, A.P. & Peel, J.S. 2003. The earliest Cambrian helcionellod mollusc *Anabarella* Vostokova. Palaeontology 46, 1073–1087.

Ivantsov, A.Yu. 1990. (First finds of phyllocarids in the Lower Cambrian of Yakutia). Paleontologicheskiy Zhurnal 1990 (2), 130–132 (in Russian).

Ivantsov, A.Yu. 2017. (On the finds of typical Ediacaran fossils in the Vendian Yudoma Group of eastern Siberia). Doklady Akademii nauk 472, 1–4 (in Russian).

Ivantsov, A.Yu., Zhuravlev, A.Yu., Krassilov, V.A., Leguta, A.V., Mel’nikova, L.M., Urbanek, A., Ushatinskaya, G.T. & Malakhovskaya, Ya.E. 2005. [Unique Sinsk Localities of Early Cambrian Organisms (Siberian Platform)]. Paleontologicheskiy Institut Rossiyskoy Academii Nauk, Trudy 284, 1–143 (in Russian).

Karlova, G.A. & Vodanyuk, S.A. 1985. [New data on transitional strata to the Cambrian in the Khorbusuonka River basin (Olenek Uplift)]. In V.V. Khomentovsky, ed. (Late Precambrian and Early Palaeozoic Stratigraphy of Siberia: Vendian and Riphean), pp. 3–13. Novosibirsk, IGiG SO AN SSSR (in Russian).

Khomentovsky, V.V. 2008. The Yudomian of Siberia, Vendian and Ediacaran systems of the International Stratigraphic Scale. Stratigraphy and Geological Correlation 16, 581–598.

Khomentovskiy, V.V. & Karlova, G.A. 1986. (On the lower boundary of the Pestrotsvet Formation in the Aldan River basin). In V.V.Khomentovskiy, ed. (Late Precambrian and Early Palaeozoic of Siberia. Siberian Platform and the Outer Zone of the Sayan-Altay Fold Belt), p. 3–22. Novosibirsk, IGiG SO AN SSSR (in Russian).

Khomentovskiy, V.V. & Karlova, G.A. 1991. (New data on a correlation of the Vendian-Cambrian strata in eastern and transitional facies regions of Yakutia). In V.V.Khomentovskiy, ed. (Late Precambrian and Early Palaeozoic of Siberia. Siberian Platform and Its Outskirts), p. 3–44. Novosibirsk, IGiG SO AN SSSR (in Russian).

Khomentovsky, V.V. & Karlova, G.A. 1992. (The Cambrian lower boundary in its grounding in Siberia). Geologiya i Geofizika 1992 (4), 3–26 (in Russian).

Khomentovsky, V.V. & Karlova, G.A. 1993. Biostratigraphy of the Vendian–Cambrian beds and the lower Cambrian boundary in Siberia. Geological Magazine 130, 29–45.

Khomentovsky, V.V. & Karlova, G.A. 2002. The boundary between Nemakit-Daldynian and Tommotian stages (Vendian–Cambrian systems) of Siberia. Stratigraphy and Geological Correlation 10, 217–238.

Khomentovskii, V.V. & Karlova, G.A. 2005. The Tommotian Stage base as the Cambrian lower boundary in Siberia. Stratigraphy and Geological Correlation 13, 21–34.

Khomentovsky, V.V. & Repina, L.N. 1965. (The Lower Cambrian of the Stratotype Section of Siberia). Moscow, Nauka. 200 p. (in Russian).

Khomentovsky, V.V., Shenfil’, V.Yu., Yakshin, M.S. & Butakov, S.P. 1972. (The reference sections of the Upper Precambrian and Lower Cambrian strata on the Siberian Platform). Institut Geologii i Geofiziki Sibirskogo Otdeleniya Akademii Nauk SSSR, Trudy 141, 1–356 (in Russian).

Khomentovsky, V.V., Didenko, A.N. & Pyatiletov, V.G. 1982. (General conclusions on the Vendian stratigraphy in the western Prianabar’e). In V.V. Khomentovsky, ed. (New Data on the Late Precambrian Stratigraphy of Siberia), pp. 3–20. Novosibirsk, IGiG SO AN SSSR (in Russian).

Khomentovsky, V.V., Val’kov, A.K., Karlova, G.A. & Nuzhnov, S.V. 1983. (Key section of the Precambrian-Cambrian strata of the Gonam River). In V.V. Khomentovsky, ed. (Late Precambrian and Early Palaeozoic of Siberia. Vendian Strata), pp. 29–44. Novosibirsk, IGiG SO AN SSSR (in Russian).

Khomentovsky, V.V., Val’kov, A.K. & Karlova, G.A. 1991. (New data on the biostratigraphy of transitional Vendian–Cambrian strata in the middle reaches of the Aldan River). In V.V. Khomentovsky & A.S. Gibsher, eds. (Late Precambrian and Early Palaeozoic of Siberia. Problems of Regional Stratigraphy), pp. 3–57. Novosibirsk, IGiG SO AN SSSR (in Russian).

Kochnev, B.B. & Karlova, G.A. 2005. New data on biostratigraphy of the Vendian Nemakit-Daldynian Stage in the southern Siberian Platform. Stratigraphy and Geological Correlation 18, 492–504.

Kokoulin, M.L., Zinchenko, V.N., Vasil’eva, N.I., Zazhigin, S.V., Rudavskaya, V.A. & Burova, I.A. 1991. (Key section of Vendian and Cambrian strata of the Yakutian Uplift). In Yu.L. Slastenov, ed. (Regional Geology and Mineral Resources of Yakutia), pp. 3–15. Yakutsk, Yakutskoe Knizhnoe Izdatel’stvo (in Russian).

Kontorovich, A.E., Varlamov, A.I., Grazhdankin, D.V., Karlova, G.A., Klets, A.G., Kontorovich, V.A., Saraev, S.V., Terleev, A.A., Belyaev, S.Yu., Varaksina, I.V., Efimov, A.S., Kochnev, B.B., Nagovitsin, K.E., Postnikov, A.A. & Filippov, Yu.F. 2008. A section of Vendian in the east of West Siberian Plate (based on data from the Borehole Vostok 3). Russian Geology and Geophysics 49, 932–939.

Korde, K.B. 1959. (Problematic fossils from Cambrian strata of the southeastern Siberian Platform). Doklady Akademii Nauk SSSR 125, 625–627 (in Russian).

Korobov, M.N. 1963. (New trilobites from the Lower Cambrian of the Kharaulakh Mountains). Paleontologicheskiy Zhurnal 1963 (4), 64–75 (in Russian).

Korobov, M.N. 1966a. (New trilobites from the Lower Cambrian of Yakutia). Paleontologicheskiy Zhurnal 1966 (2), 57–66 (in Russian).

Korobov, M.N. 1966b. (New trilobites of the family Conocoryphidae from the Cambrian of the Siberian Platform). Paleontologicheskiy Zhurnal 1966 (4), 92–97 (in Russian).

Korobov, M.N. 1973. (Trilobites of the family Conocoryphidae and their significance for the stratigraphy of Cambrian strata). Geologicheskiy Institut Akademii Nauk SSSR, Trudy 211, 1–161 (in Russian).

Korovnikov, I.V. 1998. Early and Middle Cambrian phylogeny of Acrothelidae brachiopods. Russian Geology and Geophysics 39, 94–99.

Korovnikov, I.V. 2001. Lower and Middle Cambrian boundary and trilobites from northeast Siberian Platform. Palaeoworld 13, 270–275.

Korovnikov, I.V. 2002. New data on biostratigraphy of the Lower and Middle Cambrian Series in the northeastern Siberian Platform. Russian Geology and Geophysics 43, 826–836.

Korovnikov, I.V. 2006. Lower-Middle Cambrian boundary in open shelf facies of the Siberian Platform. Palaeoworld 15, 424–430.

Korovnikov, I.V. 2007. Trilobites of the suborder Eodiscina from the Lower Cambrian of the northeastern Siberian Platform (Khorbosuonka River section). Paleontological Journal 41, 614–620.

Korovnikov, I.V. 2011. The lower boundary of the Toyonian stage (Cambrian) of the Siberian Platform. Russian Geology and Geophysics 52, 717–724.

Korovnikov, I.V. & Novozhilova, N.V. 2012. New biostratigraphical constraints on the Lower and lower Middle Cambrian of the Kharaulakh Mountains (northeastern Siberian Platform, Chekurovka anticline). Russian Geology and Geophysics 53, 776–786.

Korovnikov, I.V. & Shabanov, Yu.Ya. 2008. [Lingulates (brachiopods) from the Middle Cambrian of southeastern Prianabar’e (borehole KCC-2, 519)]. Novosti Paleontologii i Stratigrafii 2008 (10-11), 132–137 (in Russian).

Korovnikov, I.V. & Shabanov, Yu.Ya. 2016. Trilobites and biostratigraphy of the Kuonamka Formation, northern Siberian Platform (Olenek River). Russian Geology and Geophysics 57, 562–573.

Korovnikov, I.V., Rowland, S.M., Luchinina, V.A., Shabanov, Yu.Ya. & Fedoseev, A.V. 2002. Biostratigraphy of the Vendian, Lower and Middle Cambrian section of the Enisey River in the Plakhinskiy Island area (north-west of the Siberian Platform). Russian Geology and Geophysics 43, 334–342.

Korshunov, V.I. 1968. (*Gonamispongia*—new sponge genus of the family Chancelloriidae). Paleontologicheskiy Zhurnal 1968 (3), 127–129 (in Russian).

Korshunov, V.I. 1972. (Lower Cambrian Biostratigraphy and Archaeocyaths of the Northeastern Aldan Anteclise). Yakutsk, Yakutskoe Knizhnoe Izdatel’stvo. 128 p. (in Russian).

Korshunov, V.I. & Zhuravleva, I.T. 1967. (New archaeocyath species from the Lower Cambrian of Yakutia). In A.B. Ivanovskiy & B.S. Sokolov, eds. (New Data on the Lower Palaeozoic Biostratigraphy of the Siberian Platform), p. 3–11. Novosibirsk, Nauka (in Russian).

Kouchinsky, A. 2000. Shell microstructure in early Cambrian molluscs. Acta Palaeontologica Polonica 45, 119–150.

Kouchinsky, A. & Bengtson, S. 2017. X-ray tomographic microscopy tightens affinity of the early Cambrian *Oymurania* to the brachiopod stem group. Acta Palaeontologica Polonica 62, 39–43.

Kouchinsky, A., Bengtson, S., Pavlov, V., Runnegar, B., Val’kov, A. & Young, E. 2005. Pre-Tommotian age of the lower Pestrotsvet Formation in the Selinde section on the Siberian Platform: Carbon isotope evidence. Geological Magazine 142, 319–325.

Kouchinsky, A., Bengtson, S., Pavlov, V., Runnegar, B., Torssander, P., Young, E. & Ziegler, K. 2007. Carbon isotope stratigraphy of the Precambrian–Cambrian Sukharikha River section, northwestern Siberian platform. Geological Magazine 144, 1–10.

Kouchinsky, A., Bengtson, S., Feng, W., Kutygin, R. & Val’kov, A. 2009. The Lower Cambrian fossil Anabaritids: affinities, occurrences and systematics. Journal of Systematic Palaeontology 7, 241–298.

Kouchinsky, A., Bengtson, S. & Murdock, D.E.J. 2010. A new tannuolinid problematic from the lower Cambrian of the Sukharikha River in northern Siberia. Acta Palaeontologica Polonica 55, 321–331.

Kouchinsky, A., Bengtson, S., Clausen, S., Gubanov, A., Malinky, J.M. & Peel, J.S. 2011. A middle Cambrian fauna of skeletal fossils from the Kuonamka Formation, northern Siberia. Alcheringa 35, 123–189.

Kouchinsky, A., Bengtson, S., Runnegar, B., Skovsted, C., Steiner, M. & Vendrasco, M. 2012. Chronology of early Cambrian biomineralization. Geological Magazine 149, 221–251.

Kouchinsky, A., Bengtson, S., Clausen, S. & Vendrasco, M.J. 2015. An early Cambrian fauna of skeletal fossils from the Emyaksin Formation, northern Siberia. Acta Palaeontologica Polonica 60, 421–512.

Kouchinsky, A., Bengtson, S., Landing, E., Steiner, M., Vendrasco, M. & Ziegler, K. 2017. Terreneuvian stratigraphy and faunas from the Anabar Uplift, Siberia. Acta Palaeontologica Polonica 62, 311–440.

Kras’kov, L.N., Lazarenko, N.P., Ogienko, L.V. & Chernysheva N.E. 1960. (New Early Palaeozoic trilobites of eastern Siberia and Kazakhstan). In B.P. Markovskiy, ed. (New Species of Ancient Plants and Invertebrates of the USSR, Part II), pp. 211–256. Moscow, Gostoptekhizdat (in Russian).

Kruse, P.D., Zhuravlev, A.Yu. & James, N.P. 1995. Primordial metazoan-calcimicrobial reefs: Tommotian (Early Cambrian) of the Siberian Platform. Palaios 10, 291–321.

Landing, E. & Kouchinsky, A. 2016. Correlation of the Cambrian Evolutionary Radiation: geochronology, evolutionary stasis of earliest Cambrian (Terreneuvian) small shelly fossil (SSF) taxa, and chronostratigraphic significance. Geological Magazine. doi: 10.1017/S0016756815001089

Lazarenko, N.P. 1954. (On some Middle Cambrian trilobites of Siberia). Vestnik LGU, Seriya Biologiya, Geografiya i Geologiya 1954 (4), 153–164 (in Russian).

Lazarenko, N.P. 1957. (New data on trilobites of the genus *Triangulaspis*). In (Collection of Papers on Palaeontology and Biostratigraphy, Issue 3), pp. 3–17. Leningrad, NIIGA (in Russian).

Lazarenko, N.P. 1958. (On the find of *Bathynotus* in the Cambrian deposits of the north of the Siberian Platform). In (Collection of Papers on Palaeontology and Biostratigraphy, Issue 8), pp. 15–19. Leningrad, NIIGA (in Russian).

Lazarenko, N.P. 1962. (New Lower Cambrian trilobites from the Soviet Arctic). In N.A. Shvedov, ed. (Collection of Papers on Palaeontology and Biostratigraphy, Issue 29), pp. 29–78. Leningrad, NIIGA (in Russian).

Lazarenko, N.P. 1964. (Complexes of Lower Cambrian trilobites from the northern part of central Siberia). Nauchno-Issledovatel’skiy Institut Geologii Arktiki, Trudy 137, 166–287 (in Russian).

Lermontova, E.V. 1940. (Class Trilobita). In A.G. Vologdin, ed. (Atlas of the leading forms of the fossil faunas of the USSR. Volume 1 Cambrian), pp. 112–193, pl. 35–49. Moscow; Leningrad, Gosudarstvennoe Izdatel’stvo Geologicheskoy Literatury (in Russian).

Lermontova, E.V. 1951. (Lower Cambrian Trilobites and Brachiopods from Eastern Siberia). Moscow, VSEGEI. 222 p. (in Russian).

Lysova, L.A., Galimova, B.S., Titorenko, T.N. & Fayzulina, Z.K. 1966. (Palaeontological characteristics of the Lower Cambrian strata penetrated by the Markovo reference borehole). In (Geology and Oil-Gas Capacity of Eastern Siberia), pp. 345–356. Moscow, Nedra (in Russian).

Malakhovskaya, Ya.E. 2013. Morphogenesis and evolution of *Kutorgina* Billings, 1861 (Brachiopoda, Kutorginida). Paleontological Journal 47, 11–22.

Melnikova, L.M., Siveter, D.J. & Williams, M. 1997. Cambrian Bradoriida and Phosphatocopida (Arthropoda) of the former Soviet Union. Journal of Micropalaeontology 16, 179–191.

Meshkova, N.P. 1974. (Lower Cambrian hyoliths of the Siberian Platform). Institut Geologii i Geofiziki Sibirskogo Otdeleniya Akademii Nauk SSSR, Trudy 97, 1–110 (in Russian).

Meshkova, N.P., ed. 1983. (Lower and Middle Cambrian biostratigraphy and palaeontology of northern Asia). Institut Geologii i Geofiziki Sibirskogo Otdeleniya Akademii Nauk SSSR, Trudy 541, 1–210 (in Russian).

Meshkova, N.P. & Nikolaeva, I.V., eds. 1981. [Precambrian and Cambrian boundary beds of the Siberian Platform (biostratigraphy, palaeontology, depositional conditions)]. Institut Geologii i Geofiziki Sibirskogo Otdeleniya Akademii Nauk SSSR, Trudy 475, 1–201 (in Russian).

Missarzhevsky, V.V. 1966. (First finds of *Lapworthella* in the Lower Cambrian of the Siberian Platform). Paleontologicheskiy Zhurnal 1966 (1), 13–18 (in Russian).

Missarzhevsky, V.V. 1977. [Conodonts (?) and phosphatic problematica from the Cambrian of Mongolia and Siberia]. In L.P. Tatarinov, ed. (Invertebrates of the Palaeozoic of Mongolia), p. 10–19. Nauka, Moscow (in Russian).

Missarzhevsky, V.V. 1980. [On the Cambrian and Precambrian boundary beds on the western slope of the Olenek Uplift (Olenek River)]. Byulleten’ Moskovskogo Obshchestva Ispytateley Prirody, Otdel Geologicheskiy 55, 23–34 (in Russian).

Missarzhevsky, V.V. 1989. (The oldest skeletal fossils and stratigraphy of the Precambrian–Cambrian boundary beds). Geologicheskiy Institut Akademii Nauk SSSR, Trudy 443, 1–237 (in Russian).

Missarzhevsky, V.V. & Grigor’eva, N.V. 1981. (New representatives of the order Tommotiida). Paleontologicheskiy Zhurnal 1981 (4), 91–97 (in Russian).

Nagovitsin, K.E., Rogov, V.I., Marusin, V.V., Karlova, G.A., Kolesnikov, A.V., Bykova, N.V. & Grazhdankin, D.V. 2015. Revised Neoproterozoic and Terreneuvian stratigraphy of the Lena-Anabar Basin and north-western slope of the Olenek Uplift, Siberian Platform. Precambrian Research 270, 226–245.

Naimark, E., Shabanov, Yu. & Korovnikov, I. 2011. Cambrian trilobite *Ovatorycocara* Tchernysheva, 1962 from Siberia. Bulletin of Geosciences 86, 405–422.

Netskaya, A.I. & Ivanova, V.A. 1956. (The first find of an ostracod in the Lower Cambrian of eastern Siberia). Doklady AN SSSR 111, 1095–1097 (in Russian).

Ogienko, L.V. 1991. (Middle Cambrian biostratigraphy and trilobites of the Daldyn-Alakit area of Yakutia). In G.L. Mitrofanov, ed. (Stratigraphy and Biostratigraphy of the South of Eastern Siberia), pp. 14–37. Irkutsk, Vostochno-Sibirskiy Nauchno-Issledovatel’skiy Institut Geologii, Geofiziki i Mineral’nogo Syr’ya (in Russian).

Ogienko, L.V. & Garina, S.Y. 2001. (Stratigraphy and Trilobites of the Cambrian of the Siberian Platform). Moscow, Nauchniy Mir. 380 p. (in Russian).

Ogienko, L.V., Byaliy, V.I. & Kolosnitsyna, G.R. 1974. (Biostratigraphy of the Cambrian and Ordovician Deposits of the South of the Siberian Platform). Moscow, Nedra. 207 p. (in Russian).

Palmer, A.R. & Repina, L.N. 1990. Through a glass darkly: Taxonomy, phylogeny, and biostratigraphy of the Olenellina. University of Kansas Paleontological Contributions, New series 3, 1–35.

Parkhaev, P.Yu. 2005. Two new species of the Cambrian helcionelloid mollusks from the northern part of the Siberian Platform. Paleontological Journal 39, 615–619.

Parkhaev, P.Yu. 2006a. [Adaptive radiation of Cambrian helcionelloid mollusks (Gastropoda, Archaeobranchia)]. In S.V. Rozhnov, ed. (The Evolution of Biosphere and Biodiversity), pp. 282–296. Moscow, Tovarishchestvo nauchnykh izdaniy KMK (in Russian).

Parkhaev, P.Yu. 2006b. On the genus *Auricullina* Vassiljeva, 1998 and shell pores of the Cambrian helcionelloid mollusks. Paleontological Journal 40, 20–33.

Parkhaev, P.Yu. 2006c. New data on the morphology of ancient gastropods of the genus *Aldanella* Vostokova, 1962 (Archaeobranchia, Pelagielliformes). Paleontological Journal 40, 244–252.

Parkhaev, P.Yu. 2013. *Carinopelta* nom. nov. and Carinopeltidae nom. nov.—new substitute names for a genus and family of Cambrian gastropods. Paleontological Journal 47, 454.

Parkhaev, P.Yu. 2017a. *Davidonia* nom. nov.—a new substitute name for a genus of Cambrian gastropods. Paleontological Journal 51, 574.

Parkhaev, P.Yu. 2017b. Origin and early evolution of the phylum Mollusca. Paleontological Journal 51, 91–112.

Parkhaev, P.Yu. & Karlova, G.A. 2011. Taxonomic revision and evolution of Cambrian molluscs of the genus *Aldanella* Vostokova, 1962 (Gastropoda: Archaeobranchia). Paleontological Journal 45, 1145–1205.

Parkhaev, P.Yu., Karlova, G.A. & Rozanov, A.Yu. 2012. Stratigraphic distribution of two potential index species for the GSSP of Cambrian Stage 2 – *Aldanella attleborensis* and *Watsonella crosbyi*. In Y. Zhao, M. Zhu, J. Peng, R.R. Gaines & R.L. Parsley, eds. Cryogenian – Ediacaran to Cambrian Stratigraphy and Paleontology of Guizhou, China. Journal of Guizhou University, Natural Sciences, 179–180.

Pegel’, T.V. 1984. (New trilobites from the Lower Cambrian of Siberia). In V.S. Surkov, ed. (New Species of Ancient Invertebrates and Plants from the Oil and Gas Provinces of Siberia), pp. 15–19. Novosibirsk, SNIIGGiMS (in Russian).

Pegel’, T.V. 1987. (Trilobites of the Middle Cambrian Tangha-Ust’Mil’ reefal complex of the Siberian Platform). In S.P. Bulynnikova & I.G. Klimova, eds. (New Species of Ancient Plants and Invertebrates from the Phanerozoic of Siberia), pp. 75–79. Novosibirsk, SNIIGGiMS (in Russian).

Pegel, T.V. 2000. Evolution of trilobite biofacies in Cambrian basins of the Siberian Platform. Journal of Paleontology 74, 1000–1019.

Pegel’, T.V. & Khramova, A.P. 1985. (Trilobites of the Cambrian Chukuka lithofacies complex of the Siberian Platform). In V.A. Astashkin, ed. (Stratigraphy and Palaeontology of the Precambrian and Phanerozoic of Siberia), pp. 37–46. Novosibirsk, SNIIGGiMS (in Russian).

Pegel’, T.V., Еgorovа, L.I., Shabanov, Yu.Ya., Korovnikov, I.V., Luchinina, V.А., Salikhova, А.К., Sundukov, V.М., Fedorov, А.B., Zhuravlev, А.Yu., Parkhaev, P.Yu., Demidenko, Yu.Е. 2016. (Stratigraphy of Oil and Gas Basins of Siberia. Cambrian of Siberian Platform. V. 2 – Paleontology). Novosibirsk, IPGG SB RAS. 344 p. (in Russian).

Pel’man, Yu.L. 1977. (Early and Middle Cambrian inarticulate brachiopods of the Siberian Platform). Institut Geologii i Geofiziki Sibirskogo Otdeleniya Akademii Nauk SSSR, Trudy 316, 1–168 (in Russian).

Pel’man, Yu.L., Aksarina, N.A., Koneva, S.P. Popov, L.E., Sobolev, L.P. & Ushatinskaya, G.T. 1992. (The oldest brachiopods from the territory of northern Eurasia). Novosibirsk, OIGGiM SO RAN. 145 p. (in Russian).

Pokrovskaya, N.V. 1954. (Stratigraphy of the Cambrian sediments in the south of the Siberian Platform). In N.S. Shatskiy, ed. (Problems of Geology in Asia, Volume 1), pp. 444–465. Moscow, Izdatel’stvo AN SSSR (in Russian).

Pokrovskaya, N.V. 1958. (Middle Cambrian agnostids of Yakutia). Geologicheskiy Institut Akademii Nauk SSSR, Trudy 16, 1–96 (in Russian).

Repina, L.N. 1966. [Lower Cambrian Trilobites from the South of Siberia (Superfamily Redlichioidea, Part I)]. Moscow, Nauka. 203 p. (in Russian).

Repina, L.N. 1969. [Lower and Middle Cambrian Trilobites from the South of Siberia (Superfamily Redlichioidea, Part II)]. Moscow, Nauka. 109 p. (in Russian).

Repina, L.N. 1990. (Evolution of trilobites at the early stages of their historical development). Institut Geologii i Geofiziki Sibirskogo Otdeleniya Akademii Nauk SSSR, Trudy 764, 34–44 (in Russian).

Repina, L.N., ed. 1990. (Cambrian Biostratigraphy and Palaeontology of Northern Asia). Novosibirsk, Nauka. 222 pp. (in Russian).

Repina, L.N. & Suvorova, N.P., eds. 1983. (Biostratigraphy and fauna of the Lower and Middle Cambrian boundary beds in Siberia). Institut Geologii i Geofiziki Sibirskogo Otdeleniya Akademii Nauk SSSR, Trudy 548, 1–136 (in Russian).

Repina, L.N., Lazarenko, N.P., Meshkova, N.P., Korshunov, V.T., Nikiforov, N.T. & Aksarina, N.A. 1974. [Biostratigraphy and fauna of the Lower Cambrian of the Kharulakh (Tuora-Sis Ridge)]. Institut Geologii i Geofiziki Sibirskogo Otdeleniya Akademii Nauk SSSR, Trudy 235, 1–299 (in Russian).

Riding, R. & Zhuravlev, A.Yu. 1995. Structure and diversity of oldest sponge-microbe reefs: Lower Cambrian, Aldan River, Siberia. Geology 23, 649–652.

Rowland, S.M., Luchinina, V.A., Korovnikov, I.V., Sipin, D.P., Tarletskov, A.I. & Fedoseev, A.V. 1998. Biostratigraphy of the Vendian-Cambrian Sukharikha River section, northwestern Siberian Platform. Canadian Journal of Earth Sciences 35, 339–352.

Rogov, V.I., Karlova, G.A., Marusin, V.V., Kochnev, B.B., Nagovitsin, K.E. & Grazhdankin, D.V. 2015. Duration of the first biozone in the Siberian hypostratotype of the Vendian. Russian Geology and Geophysics 56, 573–583.

Rozanov, A.Yu. 1973. (Regularities in the morphological evolution of archaeocyaths and problems of Lower Cambrian stage division). Geologicheskiy Institut Akademii Nauk SSSR, Trudy 241, 1–164 (in Russian).

Rozanov, A.Yu. & Missarzhevsky, V.V. 1966. (Biostratigraphy and fauna of the Cambrian lower horizons). Geologicheskiy Institut Akademii Nauk SSSR, Trudy 148, 1–127 (in Russian).

Rozanov, A.Yu. & Sokolov, B.S., eds. 1984. (Lower Cambrian Stage Subdivision. Stratigraphy). Moscow, Nauka. 184 p. (in Russian).

Rozanov, A.Yu. & Zhuravlev, A.Yu. 1992. The Lower Cambrian fossil record of the Soviet Union. In J.H. Lipps, & P.W. Signor, eds. Origin and Early Evolution of the Metazoa, pp. 205–282. New York, Plenum Press.

Rozanov, A.Yu., Missarzhevsky, V.V., Volkova, N.A., Voronova, L.G., Krylov, I.N., Keller, B.M., Korolyuk, I.K., Lendzion, K., Michniak, R., Pykhova, N.G. & Sidorov, A.D. 1969. (The Tommotian Stage and the Cambrian lower boundary problem). Geologicheskiy Institut Akademii Nauk SSSR, Trudy 206, 1–380 (in Russian).

Rozanov, A.Yu., Parkhaev, P.Yu., Demidenko, Yu.E., Karlova, G.A., Korovnikov, I.V., Shabanov, Yu.Ya., Ivantsov, A.Yu., Luchinina, V.A., Malakhovskaya, Ya.E., Melnikova, L.M., Naimark, E.B., Ponomarenko, A.G., Skorlotova, N.A., Sundukov, V.M., Tokarev, D.A., Ushatinskaya, G.T. & Kipriyanova, L.K. 2010. (Fossils from the Lower Cambrian Stage Stratotypes). Moscow, PIN RAN. 228 p. (in Russian).

Rozanov, A.Yu., Repina, L.N., Apollonov, M.K., Shabanov, Yu.Ya., Zhuravlev, A.Yu., Pegel’, T.V., Fedorov, A.B., Astashkin, V.A., Zhuravleva, I.T., Egorova, L.I., Chugaeva, M.N., Dubinina, S.V., Ermak, V.V., Esakova, N.V., Sundukov, V.V., Sukhov, S.S. & Zhemchuzhnikov, V.G. 1992. (The Cambrian of Siberia). Novosibirsk, Nauka. 135 p. (in Russian).

Rozhnov, S.V. 2006. Carpozoan echinoderms from the Middle Cambrian (Mayaktakh Formation) of Siberia (lower reaches of the Lena River). Paleontological Journal 40, 266–275.

Rozhnov, S.V., Fedorov, A.B. & Sayutina, T.A. 1992. Lower Cambrian echinoderms from Russia. Paleontological Journal 1, 53–66.

Rozova, A.B. 1964. (Biostratigraphy and Descriptions of Trilobites from the Middle and Upper Cambrian of the North-West of the Siberian Platform). Moscow, Nauka. 148 p. (in Russian).

Rudavskaya, V.A. & Vasil’eva, N.I. 1985. (Acritarchs and skeletal problematics on the Vendian, Tommotian and Atdabanian stage boundaries). In M.L. Kokoulin & V.A. Rudavskaya, eds. (Late Precambrian and Early Palaeozoic Stratigraphy of the Siberian Platform), pp. 51–57. Leningrad, VNIGRI (in Russian).

Salikhova, A.K. 1987. (New Middle Cambrian trilobites of the Olenek Uplift). In S.P. Bulynnikova & I.G. Klimova, eds. (New Species of Ancient Plants and Invertebrates from the Phanerozoic of Siberia), pp. 79–85. Novosibirsk, SNIIGGiMS (in Russian).

Savitskiy, V.E. , ed. 1979. (Geology of reef systems in the Cambrian of Western Yakutia). Sibirskiy Nauchno-Issledovatel’skiy Institut Geologii, Geofiziki i Mineral’nogo Syr’ya, Trudy 270, 1–151 (in Russian).

Savitskiy, V.E., Shabanov, Yu.Ya. & Shishkin, B.B, 1964. (Stratigraphy of Lower and early Middle Cambrian deposits in the Igarka area). Sibirskiy Nauchno-Issledovatel’skiy Institut Geologii, Geofiziki i Mineral’nogo Syr’ya, Trudy 32 (2), 42–62 (in Russian).

Savitskiy, V.E., Shishkin, B.B. & Shabanov, Yu.Ya. 1967. (On the stratigraphic subdivision of the Precambrian and Cambrian deposits of the Igarka area). Sibirskiy Nauchno-Issledovatel’skiy Institut Geologii, Geofiziki i Mineral’nogo Syr’ya, Trudy, Seriya Regional’naya Geologiya 57, 133–149 (in Russian).

Savitskiy, V.E., Evtushenko, V.M., Egorova, L.L., Kontorovitch, A.E. & Shabanov, Yu.Ya. 1972. [Cambrian of the Siberian Platform (Yudoma-Olenek section type, Kuonamka complex deposits)]. Sibirskiy Nauchno-Issledovatel’skiy Institut Geologii, Geofiziki i Mineral’nogo Syr’ya, Trudy 130, 1–198 (in Russian).

Sayutina, T.A. 1980. (Early Cambrian family Khasaktiidae fam. nov.—possible stromatoporates). Paleontologicheskiy Zhurnal 1980 (4), 13–28 (in Russian).

Shabanov, Yu.Ya., Astashkin, V.A., Pegel, T.V., Egorova, L.I., Zhuravleva, I.T., Pel’man, Yu.L., Sundukov, V.M., Stepanova, M.V., Sukhov, S.S., Fedorov, A.B., Shishkin, B.B., Vaganova, N.V., Ermak, V.I., Ryabukha, K.V., Yadrenkina, A.G., Abaimova, G.P., Lopushinskaya, T.V., Sychev, O.V. & Moskalenko, T.A. 1987. [Lower Palaeozoic of the Anabar Anteclise Southwestern Slope (according to borehole data)]. Novosibirsk, Nauka, 208 pp. (in Russian).

Shabanov, Yu.Ya., Korovnikov, I.V., Pereladov, V.S., Fefelov, A.F., Lazarenko, N.P., Gogin, I.Ya., Pegel, T.V., Sukhov, S.S., Abaimova, G.P., Egorova, L.I., Fedorov, A.B., Raevskaya, E.G. & Ushatinskaya, G.T. 2008. The Cambrian System of the Siberian Platform. Part 2: North-East of the Siberian Platform. Moscow; Novosibirsk, PIN RAS. 140 pp.

Shishkin, B.B. 1974. [Shelly fauna in the Nemakit-Daldyn Formation (northwest of the Anabar uplift)]. Geologiya i Geofizika 1974 (4), 111–114 (in Russian).

Shishkin, B.B., Fedorov, A.B. & Sundukov, V.M. 1982. (Kotuy archaeocyathan horizon in the southwestern Prioanabar’e). In V.V. Khomentovsky, ed. (New Data on the Late Precambrian Stratigraphy of Siberia), pp. 20-30. Novosibirsk, IGiG SO AN SSSR (in Russian).

Skovsted, C.B., Ushatinskaya, G.T., Holmer, L.E., Popov, L.E. & Kouchinsky, A. 2015. Taxonomy, morphology, shell structure and ontogeny of *Pelmanotreta* nom. nov. from the lower Cambrian of Siberia. GFF 137, 1–8.

Sokolov, B.S. 1972. Vendian and Early Cambrian Sabelliditidae (Pogonophora) of the USSR. In Proceedings of the 23d International Geological Congress, pp. 79–86. Prague, IPU.

Sokolov, B.S. & Zhuravleva, I.T., eds., 1983. (Stage subdivision of the Early Cambrian. Atlas of fossils). Institut Geologii i Geofiziki Sibirskogo Otdeleniya Akademii Nauk SSSR, Trudy 558, 1–216 (in Russian).

Sokolov, B.S. & Zhuravleva, I.T., eds., 1985. (Problematics of the Late Precambrian and Palaeozoic). Institut Geologii i Geofiziki Sibirskogo Otdeleniya Akademii Nauk SSSR, Trudy 632, 1–178 (in Russian).

Solov’ev, I.A. 1964. (Some new trilobites from the Amgan Stage of the Olenek area of Yakutia), In N.A. Shvedov, ed. (Scientific Reports. Series Palaeontology and Biostratigraphy, Issue 4), pp. 33–55. Leningrad, NIIGA (in Russian).

Solov’ev, I.A. 1966. [On the Middle Cambrian genus *Prohedinia* (trilobites)]. In (Scientific Reports of the Scientific-Research Institute of Geology of Arctic. Palaeontology and Biostratigraphy, Issue 14), pp. 11–27. Leningrad, NIIGA (in Russian).

Solov’ev, I.A. 1969a. [New species of *Paradoxides* (trilobites) from oil shales of the Amgan Stage of northern Yakutia]. In (Scientific Reports of the Scientific-Research Institute of Geology of Arctic. Palaeontology and Biostratigraphy, Issue 25), pp. 9–20. Leningrad, NIIGA (in Russian).

Solov’ev, I.A. 1969b. (On the find of a representative of the genus *Burlingia* in the Amgan Stage of the north of Siberia), In A.A. Gerke, ed. (Scientific Reports of the Scientific-Research Institute of Geology of Arctic. Palaeontology and Biostratigraphy, Issue 26), pp. 9–12. Leningrad, NIIGA (in Russian).

Solov’ev, I.A. 1988. (New trilobites from the Lower Cambrian of the Siberian Platform). Paleontologicheskiy Zhurnal 1988 (3), 56–63 (in Russian).

Sundukov, V.M. 1983. (New archaeocyaths from the Lower Cambrian of the Lena and Kotuy). Paleontologicheskiy Zhurnal 1983 (4), 13–17 (in Russian).

Sundukov, V.M. 1984. (New species of archaeocyaths from the Lower Cambrian of the south-east of the Siberian Platform). In S.P. Bulynnikova & I.G. Klimova, eds. (New Species of Ancient Invertebrates and Plants from Oil-Gas-Bearing Provinces of Siberia), pp. 10–15. Novosibirsk, SNIIGGiMS (in Russian).

Sundukov, V.M. 1987. (New species of archaeocyaths from the Lower Cambrian of the West-Yakutian barrier reef complex). In S.P. Bulynnikova & I.G. Klimova, eds. (New Species of Ancient Plants and Invertebrates from the Phanerozoic of Siberia), pp. 46–51. Novosibirsk, SNIIGGiMS (in Russian).

Sundukov,V.M & Zhuravlev, A.Yu. 1989. (First finds of cribricyaths in the Lower Cambrian of the Siberian Platform). Paleontologicheskiy Zhurnal 1989 (3): 101–102 (in Russian).

Suvorova, N.P. 1956. (Cambrian trilobites of the east of the Siberian Platform. Issue 1: Protolenids). Paleontologicheskiy Institut Akademii Nauk SSSR 6, Trudy, 1–182 (in Russian).

Suvorova, N.P. 1958. (New trilobites from the Lower Cambrian of Yakutia). Doklady AN SSSR 122, 917–920 (in Russian).

Suvorova, N.P. 1959. (New trilobites from the superfamilies Corynexochoidea and Redlichoidea of the Lower Cambrian Lenan Stage of Yakutia). Paleontologicheskiy Zhurnal 1959 (3), 65–77 (in Russian).

Suvorova, N.P. 1960. (Cambrian trilobites of the east of the Siberian Platform. Issue 2: Olenellids-granulariids). Paleontologicheskiy Institut Akademii Nauk SSSR 84, Trudy, 1–238 (in Russian).

Suvorova, N.P. 1981. (Systematic position of some Siberian trilobites). Paleontologicheskiy Zhurnal 1981 (2), 99–109 (in Russian).

Suvorova, N.P. 1964. (Corynexochoid trilobites and their historical development). Paleontologicheskiy Institut Akademii Nauk SSSR 103, Trudy, 1–319 (in Russian).

Sysoev, V.A. 1959a. (Hyoliths of the genus *Circotheca* from the Lower Cambrian of the Taymyr District). Paleontologicheskiy Zhurnal 1959 (1), 84–92 (in Russian).

Sysoev, V.A. 1959b. (Hyoliths of the genera *Circotheca, Orthotheca* from the Lower Cambrian of the Siberian Platform). Paleontologicheskiy Zhurnal 1959 (2), 68–78 (in Russian).

Sysoev, V.A. 1962. (Cambrian Hyoliths from the Northern Slope of the Aldan Shield). Yakutsk, YaFAN SSSR. 66 p. (in Russian).

Sysoev, V.A. 1963. (Hyoliths of the genus *Torellella* from the Lower Cambrian of the Anabar Anteclise). Paleontologicheskiy Zhurnal 1963 (3), 49–55 (in Russian).

Sysoev, V.A. 1965a. (To the systematic of the order Camerothecida). In V.F. Vozin, ed. (Palaeontology and Stratigraphy of Palaeozoic and Triassic Strata of Yakutia), pp. 21–27. Moscow, Nauka (in Russian).

Sysoev, V.A. 1965b. (*Brevilabiatus*—a new hyolith genus). In V.F. Vozin, ed. (Palaeontology and Stratigraphy of Palaeozoic and Triassic Strata of Yakutia), pp. 28–30. Moscow, Nauka (in Russian).

Sysoev, V.A. 1968. (Stratigraphy and Hyoliths of the oldest Lower Cambrian strata of the Siberian Platform). Yakutsk, Yakutskoe knizhnoe izdatel’stvo. 67 p. (in Russian).

Sysoev, V.V. 1970. (On a new family of Lower Cambrian hyoliths). In A.K. Bobrov, ed. (Stratigraphy and Palaeontology of the Proterozoic and Cambrian on the East of the Siberian Platform), pp. 109–115. Yakutsk, Yakutskoe knizhnoe izdatel’stvo (in Russian).

Sysoev, V.A. 1972. (Lower Cambrian Biostratigraphy and Orthothecimorph Hyoliths of the Siberian Platform). Moscow, Nauka. 152 p. (in Russian).

Tkachenko, V.I., Ushatinskaya, G.T., Zhuravlev A.Yu. & Repina, L.N. 1987. (Cambrian strata of the Kolyma Uplift). Izvestiya Academii Nauk SSSR, Seriya Geologicheskaya 1987 (8), 55–62 (in Russian).

Ushatinskaya, G.T. 1993. (Early and Middle Cambrian lingulids of the Siberian Platform). Paleontologicheskiy Zhurnal 1993 (2), 133–136 (in Russian).

Ushatinskaya, G.T. 1994. [New Middle-Upper Cambrian acrotretids (brachiopods) from the north of the Siberian Platform and some problems of their systematic]. Paleontologicheskiy Zhurnal 1994 (4), 38–54 (in Russian).

Ushatinskaya, G.T. 1995. (The oldest lingulates). Paleontologicheskiy Institut Rossiyskoy Academii Nauk, Trudy 262, 1–91 (in Russian).

Ushatinskaya, G.T. 2012. The oldest lingulids of the Siberian Platform: Microornamentation and shell structure. Paleontological Journal 46, 1298–1308.

Ushatinskaya, G.T. & Korovnikov, I.V. 2014. Revision of the Early—Middle Cambrian Lingulida (Brachiopoda) from the Siberian Platform. Paleontological Journal 48, 28–41.

Ushatinskaya, G.T. & Korovnikov, I.V. 2016. Revision of the superfamily Acrotheloidea (Brachiopoda, class Linguliformea, order Lingulida) from the Lower and Middle Cambrian of the Siberian Platform. Paleontological Journal 50, 450–462.

Ushatinskaya, G.T. & Malakhovskaya, Ya.E. 2001. Origin and development of the Cambrian brachiopod biochores. Stratigraphy and Geological Correlation 9, 540–556.

Ushatinskaya, G.T. & Malakhovskaya, Ya.E. 2006. (The first brachiopods with a carbonate skeleton: Appearance, migration, shell wall structure). In S.V. Rozhnov, ed. (The Evolution of Biosphere and Biodiversity), pp. 177–192. Moscow, Tovarishchestvo nauchnykh izdaniy KMK (in Russian).

Val’kov, A.K. 1968. (To the fauna of the Kessyuse Formation of the Lower Cambrian of the Olenek Uplift). In K.B. Mokshantsev, ed. (Tectonics, Stratigraphy and Lithology of Sedimentary Formations in Yakutia) , pp. 115–119. Yakutsk, Yakutskoe knizhnoe izdatel'stvo (in Russian)

Val’kov, A.K. 1970. (Hyoliths of the Middle Cambrian Amgan Stage from the north-east of the Siberian Platform). In A.K. Bobrov, ed. (Stratigraphy and Palaeontology of the Proterozoic and Cambrian on the East of the Siberian Platform), pp. 71–90. Yakutsk, Yakutskoe knizhnoe izdatel’stvo (in Russian).

Val’kov, A.K. 1975. (Biostratigraphy and Hyoliths of the Cambrian of Northeastern Siberian Platform). Moscow, Nauka. 139 p. (in Russian).

Val’kov, A.K. 1982. [Biostratigraphy of the Lower Cambrian in the East of the Siberian Platform (Utchur-Maya Region)]. Moskva, Nauka. 92 p. (in Russian).

Val’kov, A.K. 1983. (Distribution of the oldest skeletal organisms and a correlation of the lower Cambrian boundary in the south-eastern part of the Siberian Platform). In V.V. Khomentovsky, ed. (Late Precambrian and Early Palaeozoic of Siberia. Vendian Strata), pp. 37–48, 88–90. Novosibirsk, IGiG SO AN SSSR (in Russian).

Val’kov, A.K. 1987. [Biostratigraphy of the Lower Cambrian of Eastern Siberian Platform (Yudoma-Olenek Region)]. Nauka, Moscow. 136 p. (in Russian).

Val’kov, A.K. & Bokova, A.R. 1989. (Composition and geological distribution of the Sunnaginian fossils on the northern slope of the Aldan Anteclise). Geologiya i Geofizika 1989 (7), 133 (in Russian).

Val’kov, A.K. & Karlova, G.A. 1984. (Fauna from transitional Vendian-Cambrian strata in the lower reaches of the Gonam River). In V.V. Khomentovsky, ed. (Late Precambrian and Early Palaeozoic Stratigraphy: Central Siberia), pp. 12–41. Novosibirsk, IGiG SO AN SSSR (in Russian).

Val’kov, A.K. & Sysoev, V.V. 1970. (Cambrian angustiochreids of Siberia). In A.K. Bobrov, ed. (Stratigraphy and Palaeontology of the Proterozoic and Cambrian on the East of the Siberian Platform), pp. 94–100. Yakutsk, Yakutskoe knizhnoe izdatel’stvo (in Russian).

Varlamov, A.I. & Sundukov, V.M. 1978. (Archaeocyathan-algal bioherms of the Olekma Formation of the Lower Cambrian on the Amga River). In L.N. Repina & I.T. Zhuravleva, eds. (News in the Lower Palaeozoic Stratigraphy and Palaeontology of Central Siberia), pp. 27–35. Novosibirsk, IGiG SO AN SSSR (in Russian).

Varlamov, A.I., Rozanov, A.Yu., Khomentovskiy, V.V., Shabanov, Yu.Ya., Abaimova, G.P., Demidenko, Yu.E., Karlova, G.A., Korovnikov, I.V., Luchinina, V.A., Malakhovskaya, Ya.E., Parkhaev, P.Yu., Pegel, T.V., Skorlotova, N.A., Sundukov, V.M., Sukhov, S.S., Fedorov, A.B. & Kipriyanova, L.K. 2008. The Cambrian System of the Siberian Platform. Part 1: The Aldan-Lena Region. Moscow; Novosibirsk, PIN RAS. 300 p.

Vasil’eva, N.I. 1985. [Biostratigraphy of the Cambrian lower horizons in north-eastern part of the Siberian Platform (lower Olenek River; Lena River; Udzha River)]. In M.L. Kokoulin & V.A. Rudavskaya, eds. (Late Precambrian and Early Palaeozoic Stratigraphy of the Siberian Platform), pp. 5–15. Leningrad, VNIGRI (in Russian).

Vasil’eva, N.I. 1986. (New anabaritid genus from the Lower Cambrian of the Siberian Platform). Paleontological Zhurnal 1986 (2), 103–104 (in Russian).

Vasil’eva, N.I. 1990. (New Early Cambrian gastropods of the Siberian Platform). In A.I. Nikolaev, ed. (Microfauna of the USSR. Problems of the Systematics and Biostratigraphy), pp. 4–21. Leningrad, VNIGRI (in Russian).

Vasil’eva, N.I. 1994. (Early Cambrian small shelly fauna from boreholes of western Yakutia). Paleontological Zhurnal 1994 (4), 3–9 (in Russian).

Vasil’eva, N.I. 1998. (Small Shelly Fauna and Biostratigraphy of the Lower Cambrian of the Siberian Platform). St.Petersburg, VNIGRI. 139 p. (in Russian).

Vasil’eva, N.I. & Rudavskaya, V.A. 1991. [Regularities in the distribution of fauna and phytoplankton communities in the Vendian and Cambrian boundary beds on the Siberian Platform). In M.S. Messezhnikov & S.A. Chirva, eds. (Methodical Aspects of Stratigraphical Studies in Oil-Gas-Bearing Basins), pp. 69–79. Leningrad, VNIGRI (in Russian).

Vasil’eva, N.I. & Sayutina, T.A. 1993. (New genus and species names for Early Cambrian chancelloriid sclerites). Paleontological Zhurnal 1993 (1), 113–114 (in Russian).

Vodanyuk, S.A. & Karlova, G.A. 1988. (On the Kessyuse Formation of the Olenek Uplift). In V.V. Khomentovsky & V.Yu. Shenfil’, eds. (Late Precambrian and Early Palaeozoic of Siberia: Riphean and Vendian), pp. 3–20. Novosibirsk, IGiG SO AN SSSR (in Russian).

Voronin, Yu.I. 1979. (Ajacicyathids of the USSR). Paleontologicheskiy Institut Akademii Nauk SSSR, Trudy 176, 1–148 (in Russian).

Voronova, L.G. & Missarzhevsky, V.V. 1969. (Finds of algae and worm tubes in the Cambrian and Precambrian boundary strata on the north of the Siberian Platform). Doklady Akademii Nauk SSSR 184, 204–210 (in Russian).

Vostokova,V.A. 1962. (The Cambrian gastropods from Siberia and Taimyr). In N.A. Shvedov, ed. (Collection of Papers on Palaeontology and Biostratigraphy, Issue 28), pp. 51–74. Leningrad, NIIGA (in Russian).

Whittington, H.B., Chatterton, B.D.E., Speyer, S.E., Fortey, R.A., Owens, R.M., Chang, W.T., Dean, W.T., Jell, P.A., Laurie, J.R., Palmer, A.R., Repina, L.N., Rushton, A.W.A., Shergold, J.H., Clarkson, E.N.K., Wilmot, N.V. & Kelly, S.R.A. 1997. Treatise on Invertebrate Paleontology, Part O (Revised), Trilobita, Introduction, Order Agnostina, Order Redlichiida. Boulder, Colorado, Geological Society of America; Lawrence, Kansas, University of Kansas. 530 p.

Williams, M., Siveter, D.J., Popov, L.E. & Vannier, J.M.C. 2007. Biogeography and affinities of bradoriid arthropods: Cosmopolitan microbenthos of the Cambrian seas. Palaeogeography, Palaeoclimatology, Palaeoecology 248, 202–232.

Zhu, M., Zhuravlev, A.Yu., Wood, R.A., Zhao, F. & Sukhov, S.S. 2017. A deep root for the Cambrian Explosion: Implications of new bio- and chemostratigraphy from the Siberian Platform. Geology . doi: 10.1130/G38865.1.

Zhuravlev, A.Yu. 2001a. Biota diversity and structure during the Neoproterozoic-Ordovician transition. In A.Yu. Zhuravlev & R. Riding, eds. The Ecology of the Cambrian Radiation, pp. 173–199. New York, Columbia University Press.

Zhuravlev, A.Yu. 2001b. (Features of the diversification of organisms in the Cambrian). In A.G. Ponomarenko, A.Yu. Rozanov & M.A. Fedonkin, eds. (Ecosystem Restructures and the Evolution of the Biosphere. Issue 4), p. 174–183. Moscow, Paleontologicheskiy Institut Rossiyskoy Akademii Nauk (in Russian).

Zhuravlev, A.Yu., Debrenne, F. & Lafuste, J. 1993. Early Cambrian microstructural diversification of Cnidaria. Courier Forschungsinstitut Senckenberg 164, 365–372.

Zhuravlev, A.Yu., Naimark, E.B. & Wood, R.A. 2015. Controls on the diversity and structure of earliest metazoan communities: early Cambrian reefs from Siberia. Earth-Science Reviews 147, 18–29.

Zhuravlev, A.Yu. & Repina, L.N., eds. 1990. Guidebook for excursion on the Aldan and Lena Rivers. Siberian Platform. 3d Internat. Symp. Cambrian System. IGIG, Novosibirsk, 115 pp.

Zhuravlev, A.Yu., & Wood, R. 1995. Lower Cambrian reefal cryptic communities. Palaeontology 18, 443–470.

Zhuravlev, A.Yu. & Wood, R.A. 2008. Eve of biomineralization: Controls on skeletal mineralogy. Geology 36, 923–926.

Zhuravleva, I.T. 1960. (Archaeocyaths of the Siberian Platform). Moscow, Akademiya Nauk SSSR. 344 p. (in Russian).

Zhuravleva, I.T., ed. 1969. (Lower Cambrian Biostratigraphy and Palaeontology of Siberia and the Far East). Moscow, Nauka. 288 p. (in Russian).

Zhuravleva, I.T., ed. 1972. (Problems of Lower Cambrian Biostratigraphy and Paleontology of Siberia). Moscow, Nauka. 228 p. (in Russian).

Zhuravleva, I.T., ed. 1973. (Problems of Lower Cambrian Paleontology and Biostratigraphy of Siberia and the Far East). Novosibirsk, Nauka. 266 p. (in Russian).

Zhuravleva, I.T., ed. 1976. (Lower and Middle Cambrian Stratigraphy and Palaeontology of the U.S.S.R.). Institut Geologii i Geofiziki Sibirskogo Otdeleniya Akademii Nauk SSSR, Trudy 296, 258 р. (in Russian).

Zhuravleva, I.T., ed. 1986. (Cambrian Biostratigraphy and Palaeontology of Northern Asia). Institut Geologii i Geofiziki Sibirskogo Otdeleniya Akademii Nauk SSSR, Trudy 669, 1–230 (in Russian).

Zhuravleva, I.T. & Meshkova, N.P., eds. 1979. (Lower Cambrian Biostratigraphy and Palaeontology of Siberia). Institut Geologii i Geofiziki Sibirskogo Otdeleniya Akademii Nauk SSSR, Trudy 406, 1–160 (in Russian).

Zhuravleva, I.T. & Repina, L.N., eds. 1988. (Cambrian of Siberia and Central Asia). Institut Geologii i Geofiziki Sibirskogo Otdeleniya Akademii Nauk SSSR, Trudy 720, 1–210 (in Russian).

Zhuravleva, I.T. & Rozanov A.Yu., eds. 1974. (Lower Cambrian Biostratigraphy and Paleontology of Europe and Northern Asia). Moscow, Nauka. 312 р. (in Russian).

Zinchenko, V.N., Vasil’eva, N.I. & Rudavskaya, V.A. 1993. (Basal Cambrian strata on the north-eastern margin of the Siberian Platform). In S.A. Chirva & V.N. Zinchenko, eds. (Phanerozoic Stratigraphy of Oil-Gas-Bearing Regions of Russia), pp. 4–18. St.Petersburg, VNIGRI (in Russian).

1. **Stratigraphy, dating and correlation**

U-Pb zircon dates of Bowring et al. (1993) on lower Cambrian Olenek River section of the Siberian Platform being verified recently show that the base of the uppermost Ediacaran (lowermost Nemakit-Daldynian) *Anabarites trisulcatus* Zone is ~543.9±0.24 Ma (Rogov et al. 2015). Congruent dates of 539.4±2.9 Ma and 536.7±3.9 Ma, respectively, characterize the Fortunian strata in South China; these figures are obtained from a tuff layer interbedded with phosphorites yielding fossils of the *Anabarites trisulcatus-Protohertzina anabarica* Assemblage Zone (Compston et al. 2008; Zhu et al. 2009). This zone is broadly correlated with the *Protohertzina anabarica* Zone of the Siberian Platform which is used herein (Steiner et al. 2007).

The lower Tommotian (Cambrian Stage 2) of the Kharaulakh Mountains on the lower Lena River is estimated in age as ~534.6±0.5 Ma following Bowring et al. (1993) and the analysis of archaeocyath assemblages which occur in the strata directly overlying the fluvial conglomerate bearing volcanic clasts (Astashkin et al. 1991). This estimation is in good agreement with the age of 529.7±0.3 Ma being proved for the volcanic tuff layering immediately above the *Watsonella crosbyi* first appearance datum (FAD) and within the middle Tommotian *Rusophycus avalonensis* FAD on the Olenek River (Nagovitsin et al. 2015). A similar U-Pb zircon date of 529.7±0.3 Ma is obtained for the Stage 2 part of the Placentian Series above the *Watsonella crosbyi* Zone of Eastern Newfoundland (Isachsen et al. 1994; Landing et al. 1998). Several further dates scattering from 529.7±0.3 Ma to 526.4±5.4 Ma from tuff layers at the base of Shuijingtuo and Shiyantou formations in South China provide further constrains for the Stage 2 upper boundary (Compston et al. 2008; Okada et al. 2014). The upper Shuijingtuo Formation yields trilobites and archaeocyaths of the Cambrian Stage 3 (Yang et al. 2016) and the Shiyantou Formation is overlain by trilobite-bearing Yu’anshan Formation and underlain by the Zhujiaquing Formation yielding Stage 2 fauna (Yang et al. 2014).

Thus, the age of the Series 2/Stage 3 boundary is suggested to be about 525 Ma, and is the age tie point for the FAD of trilobites (base of the *Profallotaspis jakutensis* Trilobite Zone and of the Atdabanian Stage) on the Siberian Platform. A volcanic ash bed in Shropshire, England gives an U-Pb zircon age for the middle *Callavia* Trilobite Zone of 514.45±0.36 Ma (Harvey et al. 2011) and provides an estimation of the uppermost Atdabanian *Fansycyathus lermontovae* Archaeocyath Zone on the Siberian Platform (Zhuravlev & Riding 2001; Zhang et al. 2017). Also, Stage 3 Maotianshan Shale Member of the Yu’anshan Formation in South China contains detrital zircon populations yielding consistent Concordia age from 521.7±6.1 Ma to 519.6±5.3 Ma (Hofmann et al. 2016) and the Re-Os isotope isochron age of 522.9±8.6 Ma implies deposition of the Niutitang Formation upper member of which contains upper Stage 3 trilobites (Wei et al. 2017). These levels are correlated with lower-middle Atdabanian of Siberia (Yang et al. 2016).

In addition U-Pb zircon age for the middle *Callavia* Trilobite Zone constrains the age of the basal Botoman (Stage 4) *Bergeroniellus micmacciformis* – *Erbiella* Trilobite Zone at 514 Ma. Ash beds of 511±1.0 Ma and 509.1±0.22 Ma in age calibrate strata bearing fossils of *Geyerorodes howleyi* and *Acadoparadaxides harlani* trilobite zones of the former Avalon continent embracing Eastern Newfoundland, England, Wales and some other areas (Landing et al. 1998; Harvey et al. 2011) and, thus, sandwich the Toyonian/Amgan (Series 2/3) boundary in Siberia at 510 Ma (Sundberg et al. 2016).

Astashkin, V.A., Pegel’, T.V., Repina, L.N., Rozanov, A.Yu., Shabanov, Yu.Ya., Zhuravlev, A.Yu., Sukhov, S.S. & Sundukov, V.M. 1991. The Cambrian System on the Siberian Platform. Correlation chart and explanatory notes. International Union of Geological Sciences, Publication 27, 1–133.

Bowring, S.A., Grotzinger, J.P., Isachsen, C.E., Knoll, A.H., Pelechaty, S.M., Kolosov, P. 1993. Calibrating rates of Early Cambrian evolution. Science 261, 1293–1298.

Compston, W., Zhang, Z., Cooper, J.A., Ma, G. & Jenkins, R.J.F. 2008. Further SHRIMP geochronology on the early Cambrian of South China. Am. J. Sci. 308, 399–420.

Harvey, T.H.P., Williams, M., Condon, D.J., Wilby, P.R., Siveter, D.J., Rushton, A.W.A., Leng, M.J. & Gabbott, S.E. 2011. A refined chronology for the Cambrian succession of southern Britain. J. Geol. Soc. London.168, 705–716.

Hofmann, M.H., Li, X.H., Chen, J., MacKenzie, L.A. & Hinman, N.W. 2016. Provenance and temporal constraints of the Early Cambrian Maotianshan Shale, Yunnan Province, China. Gondwana Research 37, 348–361.

Isachsen, C.E., Bowring, S.A., Landing, E. & Samson, S.D. 1994. New constraint on the division of Cambrian time. Geology 22, 496–498.

Landing, E., Bowring, S.A., Davidek, K., Westrop, S.R., Geyer, G. & Heldmaier. W. 1998. Duration of the Early Cambrian: U-Pb ages of volcanic ashes from Avalon and Gondwana. Can. J. Earth Sci. 35, 329–338.

Nagovitsin, K.E., Rogov, V.I., Marusin, V.V., Karlova, G.A., Kolesnikov, A.V., Bykova, N.V. & Grazhdankin, D.V. 2015. Revised Neoproterozoic and Terreneuvian stratigraphy of the Lena-Anabar Basin and north-western slope of the Olenek Uplift, Siberian Platform. Precambrian Res. 270, 226–245.

Okada, Y., Sawaki, Y., Komiya, T., Hirata, T., Takahata, N., Sano, Y., Han, J. & Maruyama, S. 2014. New chronological constraints for Cryogenian to Cambrian rocks in Three Gorges, Weng’an ang Chengjiang areas, South China. Gondwana Res. 25, 1027–1044.

Rogov, V.I., Karlova, G.A., Marusin, V.V., Kochnev, B.B., Nagovitsin, K.E. & Grazhdankin, D.V. 2015. Duration of the first biozone in the Siberian hypostratotype of the Vendian. Russian Geol. Geophys. 56, 573–583.

Steiner, M., Li, G., Qian, Y., Zhu, M. & Erdtmann, B.-D. 2007. Neoproterozoic to early Cambrian small shelly fossil assemblages and a revised biostratigraphic correlation of the Yangtze Platform (China). Palaeogeography, Palaeoclimatology, Palaeoecology 254, 67–99.

Sundberg, F.A., Geyer G., Kruse P.D., McCollum L.B., Pegel’ T.V., Żylińska A. & Zhuravlev A.Yu. 2016. International correlation of the Cambrian Series 2-3, Stage 4-5 boundary interval. Australasian Palaeontological Memoirs 49, 83–124.

Wei, S., Fu, Y., Liang, H., Ge, Z., Zhou, W. & Wang, G. 2018. Re-Os geochronology of the Cambrian stage-2 and -3 boundary in Zhijin County, Guizhou Province, China. Acta Geochimica 37, 323–333.

Yang, A., Zhu, M., Zhuravlev, A.Yu., Yuan, K., Zhang, J. & Chen, Y. 2016. Archaeocyathan zonation of the Yangtze Platform: implications for regional and global correlation of lower Cambrian stages. Geological Magazine 153, 388–409.

Yang, B., Steiner, M., Li, G. & Keupp, H. 2014. Terreneuvian small shelly faunas of East Yunnan (South China) and their biostratigraphic implications. Palaeogeography, Palaeoclimatology, Palaeoecology 398, 28–58.

Zhang, X., Ahlberg, P., Babcock, L.E., Choi, D.K., Geyer, G., Gozalo, R., Hollingsworth, J.S., Li, G., Naimark, E.B., Pegel, T., Steiner, M., Wotte, T. & Zhang, Z. 2017. Challenges in defining the base of Cambrian Series 2 and Stage 3. Earth-Science Reviews 172, 124–139.

Zhu, R., Li, X., Hou, X., Pan, Y., Wang, F., Deng, C., He, H. 2009. SIMS U-Pb zircon age of a tuff layer in the Meishucun section, Yunnan, southwest China: Constraint on the age of the Precambrian-Cambrian boundary. Sci. China Ser. D—Earth Sci. 52, 1385–1392.

Zhuravlev, A.Yu. & Riding, R., eds. 2001. The Ecology of the Cambrian Radiation. New York, Columbia University Press.

1. **Quantitative data**

Lopho = Lophotrochozoans; ND = Nemakit-Daldynian, T = Tommotian; A = Atdabanian; B = Botoman; Tn = Toyonian; Am = Amgan; Ma = million years ago;

SPP. = species.

| TIME UNIT | 1 | 2 | 3 | 4 | 5 | 6 | 7 | 8 | 9 | 10 | 11 | 12 | 13 | 14 | 15 | 16 |
| --- | --- | --- | --- | --- | --- | --- | --- | --- | --- | --- | --- | --- | --- | --- | --- | --- |
| Ma | 545 | 542.5 | 540 | 537.5 | 535 | 532.5 | 530 | 527.5 | 525 | 522.5 | 520 | 517.5 | 515 | 512.5 | 510 | 508.5 |
| Zones | ND1 | ND2 | ND3 | ND4 | T1 | T2 | T3 | T4 | A1 | A2 | A3 | A4 | B1 | B2-4 | Tn | Am1 |
| stem groups | 0 | 4 | 27 | 78 | 92 | 106 | 85 | 58 | 50 | 44 | 41 | 55 | 77 | 30 | 20 | 28 |
| crown groups | 0 | 0 | 0 | 4 | 5 | 5 | 5 | 4 | 3 | 7 | 9 | 12 | 25 | 25 | 43 | 52 |
| archaeocyaths | 0 | 0 | 0 | 0 | 13 | 31 | 40 | 36 | 57 | 52 | 41 | 50 | 77 | 3 | 8 | 0 |
| trilobites | 0 | 0 | 0 | 0 | 0 | 0 | 0 | 0 | 7 | 37 | 27 | 34 | 111 | 45 | 109 | 109 |
| TOTAL SPP. | 7 | 27 | 55 | 124 | 139 | 171 | 161 | 123 | 137 | 158 | 131 | 171 | 309 | 114 | 187 | 195 |
|  |  |  |  |  |  |  |  |  |  |  |  |  |  |  |  |  |

| Zones  TIME UNIT | ND1 | ND2 | ND3 | ND4 | T1 | T2 | T3 | T4 | A1 | A2 | A3 | A4 | B1 | B2-4 | Tn | Am1 |
| --- | --- | --- | --- | --- | --- | --- | --- | --- | --- | --- | --- | --- | --- | --- | --- | --- |
| Archaeocyatha |  |  |  |  | 13 | 31 | 40 | 36 | 57 | 52 | 41 | 50 | 77 | 3 | 8 |  |
| Radiocyatha |  |  |  |  |  | 1 | 1 | 1 |  |  |  | 1 |  |  |  |  |
| Cribricyatha |  |  |  |  |  |  |  |  | 1 |  |  |  |  |  |  |  |
| Corallomorpha |  |  |  |  |  | 1 | 1 | 1 | 3 | 2 | 2 | 2 | 1 | 1 |  |  |
| Anabaritida | 2 | 10 | 11 | 24 | 5 | 6 | 8 | 2 | 1 | 1 | 1 |  |  |  |  |  |
| Hyolithelminthes |  |  | 2 | 6 | 13 | 12 | 12 | 12 | 8 | 9 | 6 | 6 | 7 | 2 |  | 1 |
| Palaeoscolecida/Xenusia |  | 1 | 1 |  |  |  | 1 |  |  | 2 | 5 | 4 | 7 | 4 | 2 | 1 |
| Trilobita |  |  |  |  |  |  |  |  | 7 | 37 | 27 | 34 | 111 | 45 | 109 | 109 |
| other Arthropoda |  |  |  |  |  |  |  | 1 | 3 | 3 | 1 | 4 | 4 | 3 | 1 | 1 |
| Halkieriida |  | 3 | 6 | 13 | 6 | 5 | 4 | 4 | 4 | 4 | 1 | 1 | 1 | 1 |  |  |
| Helcionelloida |  |  | 15 | 46 | 43 | 30 | 19 | 6 | 6 | 7 | 3 | 9 | 16 | 6 | 5 | 10 |
| Gastropoda/Bivalvia |  |  |  | 4 | 5 | 3 | 2 | 1 | 1 | 1 | 3 | 3 | 2 | 2 | 2 | 2 |
| Tommotiida |  |  |  |  | 7 | 11 | 12 | 10 | 11 | 4 | 5 | 5 | 5 |  |  |  |
| Brachiopoda |  |  |  |  | 1 | 3 | 3 | 2 | 2 | 6 | 8 | 9 | 22 | 23 | 41 | 50 |
| Hyolitha |  | 1 | 6 | 19 | 36 | 60 | 50 | 38 | 30 | 28 | 29 | 38 | 54 | 22 | 14 | 18 |
| Stenothecoida |  |  |  |  |  |  |  |  |  | 1 | 2 | 2 | 1 | 1 | 1 |  |
| Protoconodonta |  |  | 7 | 7 | 3 | 2 | 2 | 2 | 1 | 1 | 1 | 1 | 1 | 1 | 3 | 3 |
| other | 5 | 12 | 7 | 5 | 7 | 6 | 6 | 7 | 2 |  | 1 | 4 |  |  |  |  |
| TOTAL spp. | 7 | 27 | 55 | 124 | 139 | 171 | 161 | 123 | 137 | 158 | 131 | 171 | 309 | 114 | 187 | 195 |
| TOTAL Lophotrochozoa |  | 4 | 27 | 82 | 97 | 111 | 90 | 62 | 53 | 51 | 50 | 67 | 102 | 55 | 63 | 80 |
